# Out of the Crystalline Comfort Zone: Sampling the Initial Oxide Formation At Cu(111)

**DOI:** 10.1002/advs.202513878

**Published:** 2025-10-27

**Authors:** Felix Riccius, Nicolas Bergmann, Hendrik H. Heenen, Karsten Reuter

**Affiliations:** ^1^ Fritz‐Haber‐Institut der Max‐Planck‐Gesellschaft Faradayweg 4‐6 D‐14195 Berlin Germany

**Keywords:** advanced sampling, machine‐learned interatomic potentials, phase transition, statistical thermodynamics, surface oxidation

## Abstract

Oxidizing transition metal surfaces are generally characterized by an increasing heterogeneity at simultaneous lowering of crystalline order. This complexity eludes present‐day first‐principles descriptions, with predictive‐quality surface phase diagrams commonly derived from comparing the stability of a small number of ordered surface structural models that are motivated by partial experimental characterization or chemical intuition. Here the computational acceleration brought by machine‐learned interatomic potentials is leveraged for a systematic sampling of the configurational phase space through replica exchange molecular dynamics. Thermodynamic averaging subsequently yields grand‐canonical expectation values for observables like O coverage that account for the disorder and diversity of the sampled structures. Application to the initial oxidation of the Cu(111) surface reveals the (purely entropic) stabilization of sparse O adsorbates at the onset, a plethora of energetically essentially degenerate polymeric –O–Cu–O– ring and chain networks at higher O loading, as well as the presence of experimentally discussed minority species. The in silico surface phase diagram correspondingly shows marked differences to one based merely on established ordered surface reconstructions.

## Graphical abstract

By combining replica exchange molecular dynamics with fast and efficient machine‐learned interatomic potentials, this computational study predicts the complex surface reconstructions accompanying the early Cu(111) surface oxidation. The unprecedentedly exhaustive sampling results in a high‐quality surface phase diagram that not only recovers known reconstructions, but also explains the accompanying disorder and uncovers new entropically‐stabilized low‐coverage phases.

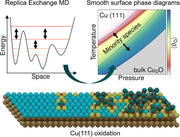

## 1 Introduction

A detailed understanding of the oxidation of transition metal surfaces is key to technologically relevant fields as diverse as optoelectronics,^[^ ^1^ ^]^ sensors,^[^ ^2^ ^]^ corrosion protection,^[^ ^3^ ^]^ or heterogeneous catalysis.^[^ ^4^, ^5^, ^6^ ^]^ To this end, decades‐long extensive experimental characterization efforts largely follow two major strands. Controlled oxygen exposure in ultra‐high vacuum (UHV) provides most resolved spectromicroscopic information, e.g., through scanning tunneling microscopy (STM), whereas in situ experiments under (near‐)ambient conditions relate to dynamic aspects and the influence of oxidation on a surface's functionality. A central insight that has emerged from the whole of such works is that surface oxidation is generally far more complex than either just on‐surface adsorption of oxygen adatoms at sub‐monolayer (ML) coverage or immediate formation of thick bulk‐like oxide overlayers.^[^ ^4^, ^7^, ^8^ ^]^ Instead, it is often nanometer thin oxidic films that prevail under environmental conditions most relevant to the specific applications.^[^ ^9^ ^]^ The atomic structure and chemical composition of these surface oxides can thereby largely deviate from any known bulk oxide crystal, with an overall much lower degree of crystallinity and larger heterogeneity of the oxidized surface fringe. In fact, this disorder and concomitant dynamics is increasingly recognized as an essential feature for the surface functionality in applications like heterogeneous catalysis.^[^ ^10^, ^11^, ^12^ ^]^

Unfortunately, experimental characterization beyond this generic understanding proves to be highly challenging, both technically and conceptually. In situ approaches still struggle with resolution or provide data too complex for atomic‐scale interpretation. At the UHV side, it is frequently difficult to relate the more specific data to application‐relevant environments, in particular as there is a prevailing preference for low‐temperature measurements. Furthermore, structural techniques are either only sensitive to or focused on crystalline domains of the surface. As an upshot, it is then fundamentally unclear whether reported ordered (surface oxide) structures are equilibrated, only kinetically stabilized, or whether they are even characteristic for the oxidized surface at all.

In this situation, predictive‐quality first‐principles calculations can in principle contribute most valuable complementary information. Especially, efficient ab initio thermodynamics (AITD) approaches^[^ ^10^, ^13^ ^]^ have been extensively employed to evaluate the stability of surface structural models in increasingly O‐rich atmospheres.^[^ ^14^ ^]^ Grand‐canonical AITD derives its efficiency primarily from considering the surrounding gas phase at the level of thermodynamic reservoirs, in the case of surface oxidation suitably expressed via an oxygen chemical potential Δ*µ*_O_ = Δ*µ*_O_(*T*, *p*) that combines the dependence on temperature *T* and oxygen pressure *p*. The high computational cost of the underlying first‐principles calculations, typically performed at the semi‐local density functional theory (DFT) level, has nevertheless restricted practical usage of AITD to a static reductionist sampling (SRS), i.e., to simply compare the relative stability of a restricted number of human‐selected and locally geometry‐optimized surface structural models with varying O‐content. Furthermore, at limited computationally tractable surface‐unit cell sizes and often inspired by experimental (e.g., STM) data, these selected models only comprise ordered structures. In consequence, resulting predicted SRS surface phase diagrams also only feature stability domains of various ordered structures as a function Δ*µ*_O_ and are in general highly dependent on the considered pool of candidate structural models.^[^ ^10^ ^]^

Recently established machine‐learned interatomic potentials (MLIPs) offer an intriguing possibility to overcome these limitations. Suitably trained, these surrogate models provide DFT‐quality energies and forces at orders of magnitude lower computational cost.^[^ ^15^, ^16^, ^17^, ^18^ ^]^ This enhanced efficiency allows for much improved sampling strategies and we here specifically perform extensive replica‐exchange molecular dynamics (REMD)^[^ ^19^, ^20^, ^21^ ^]^ in the canonical ensemble while systematically varying the surface composition. Able to perform these simulations in larger surface unit‐cells, the obtained trajectory data notably also extends to disordered structures. In a first step, we employ this data for an exploration of the configurational space and harvest a sizable pool of (meta)stable candidate structural models far beyond the biased SRS hand‐selection. Suitably averaging over the entire REMD trajectories then allows to determine expectation values for observables like the oxygen coverage, which account for vibrational and configurational entropic effects at finite temperatures.

We demonstrate the effect of this advanced AITD sampling strategy for the description of the initial oxidation of the Cu(111) surface. Extending the candidate pool from a small number of previously reported ordered surface oxide structures to the finite‐temperature REMD ensemble leads indeed to significant changes of the derived picture of the oxidizing surface. We find a considerable stabilization of dispersed O adsorbates and O‐decorated Cu adatoms.^[^ ^7^, ^22^ ^]^ The latter can be viewed as monomeric –O–Cu–O– units that coalesce into a polymeric ring and chain network upon continued oxidation. The ensemble data reveals a considerable variability and flexibility of this network structure, with a plethora of competing (disordered) structures energetically degenerate to the hitherto predominantly discussed ordered so‐called “44” and “29” –O–Cu–O– networks.^[^ ^23^, ^24^, ^25^, ^26^ ^]^ This is consistent with the large degree of structural disorder observed among these two well‐known surface oxide reconstructions in STM studies^[^ ^25^, ^27^, ^28^ ^]^ – and it makes contact to the view of the oxidizing surface as a heterogeneous ensemble of coexisting (and possibly dynamically evolving^[^ ^29^ ^]^) structures with distinct geometric motifs. The REMD ensemble provides an atomic‐scale model for the concomitant rich variety of (active) sites of the oxidizing surface. Averaging over its partition function establishes a first step to capture the effect of this diversity on macroscopic functionalities like the catalytic properties.^[^ ^30^, ^31^, ^32^ ^]^

## 2 Experimental Section

For efficient, yet accurate sampling via REMD simulations, we train an MLIP on the basis of the MACE architecture^[^ ^33^ ^]^ to DFT calculations. The latter were performed with the VASP package,^[^ ^34^, ^35^ ^]^ employing the projector‐augmented wave (PAW) method^[^ ^36^ ^]^ and a plane wave basis set in periodic boundary conditions. The Perdew–Burke–Ernzerhof (PBE) exchange‐correlation functional^[^ ^37^ ^]^ is used, which we found to describe both bulk Cu and Cu(I) oxide (Cu_2_O) reasonably well. The MLIP is trained via an active‐learning routine,^[^ ^38^, ^39^, ^40^ ^]^ where an initial training set is iteratively expanded with configurations selected from the REMD simulations that in turn are driven by the continuously re‐trained MLIP. The resulting MACE potential accurately reproduces DFT energies and forces with total root mean square errors (RMSEs) of 3 meV/atom and 148meV/Å, respectively, which results in only minor errors of e.g. bulk lattice constants of ⩽0.08 % and surface energies ≤ $1.8\;\mathrm{meV}/{Å}^{2}$ (see Sections S1.1 and S1.2, Supporting Information for further details on the DFT calculations, MLIP training, and validation).

**Figure 1:**
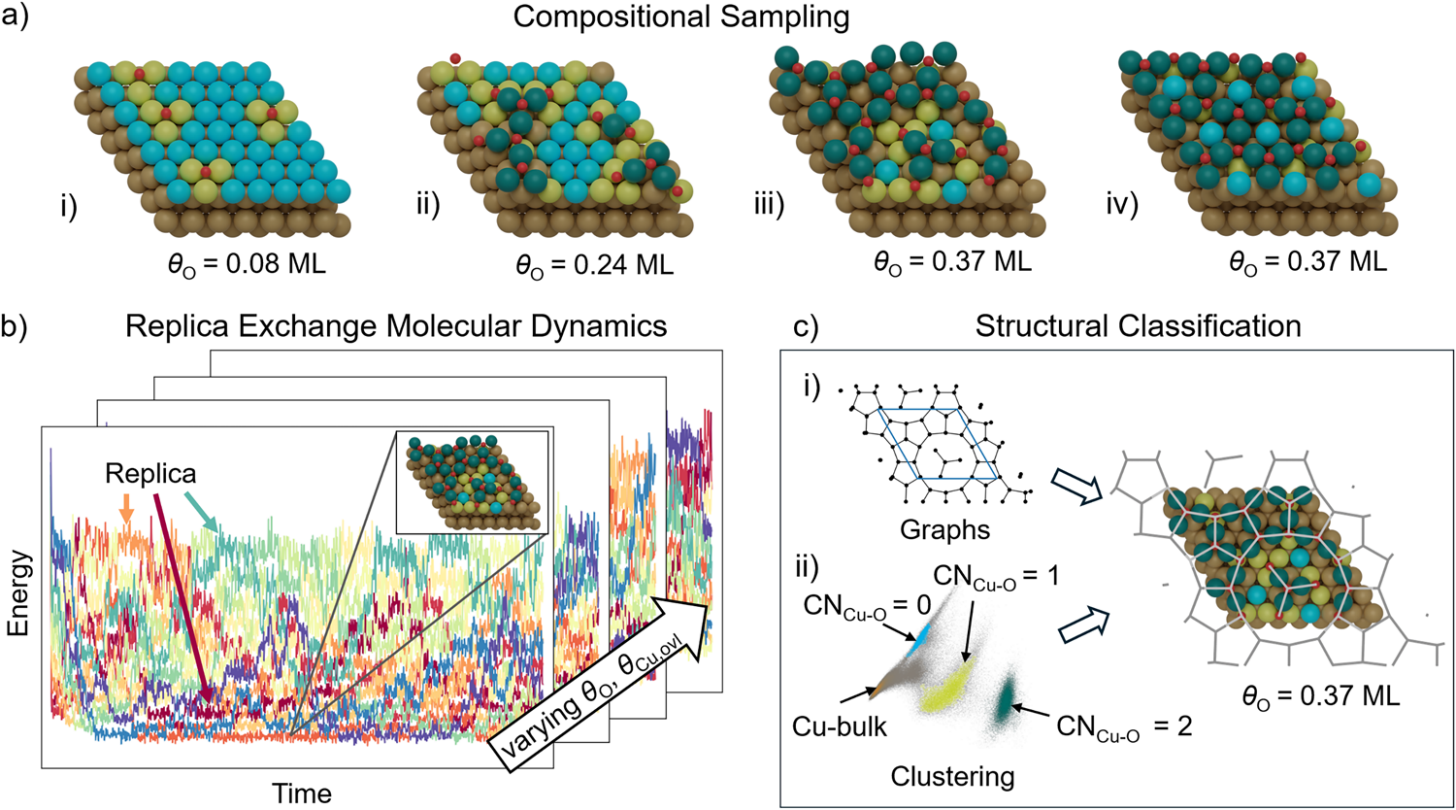
Schematic of the computational approach followed in this work. a) Top views of the employed (7 × 7)‐Cu(111) surface unit‐cell, illustrating characteristic structures accessible in the systematically investigated O coverage *θ*_O_: i) clean surface with oxygen adatoms, ii) monomeric or aggregating –O–Cu–O– units, iii) polymeric –O–Cu–O– networks exposing various ring and chain structures, and iv) a crystalline (6 × 6)‐Cu_2_O overlayer at *θ*_O_ = 0.37 monolayer (ML). Note, that the simultaneous variation of copper content in the simulation cell (*θ*_Cu, ovl_) enables the formation of disordered and crystalline structures at the same nominal *θ*_O_, as illustrated by iii) and iv). b) Energy of different replicas against simulation time in REMD simulations used to explore the canonical ensembles at the different surface compositions (*θ*_O_, *θ*_Cu, ovl_). c) Classification of different i) –O–Cu–O– rings and chains via a graph theory approach as well as ii) local Cu environments to unambiguously distinguish Cu bulk and Cu surface atoms coordinated by 0, 1, and 2 oxygen atoms in the sampled (disordered) structures, CN_Cu‐O_ = 0, 1, 2. The respectively coordinated Cu atoms are shown as orange, blue, light green, and dark green spheres, with O atoms in red.

The surface calculations are performed with asymmetric slabs, containing 4 Cu(111) layers with the bottom layer frozen to bulk configuration. The various ordered adsorbate structures and surface reconstructions discussed below are modeled as overlayers atop this core slab and in their respective surface unit‐cells (see Supporting Information for details on reciprocal space sampling). The REMD simulations assess the configuration space for the initial oxidation in a (7 × 7) surface unit‐cell, see **Figure** **1**a, and systematically consider 400 compositions with 0 ⩽ *N*_Cu, ovl_ ⩽ 48 and 0 ⩽ *N*_O_ ⩽ 30 additional Cu and O atoms at the surface of the core slab. This thus spans an oxygen coverage range *θ*_O_ from 0 to ≈ 0.61 monolayer (ML) and a Cu overlayer range *θ*_Cu, ovl_ up to another ML. At *θ*_O_ = 0.37 ML and *θ*_Cu, ovl_ = 0.73 ML, this compositional range notably allows to accommodate a coherent (6 × 6)‐Cu_2_O(111) oxide overlayer^[^ ^41^, ^42^ ^]^ at minimal 1.64–0.26% strain in the temperature range 0–900 K, respectively. For each composition, the LAMMPS code^[^ ^43^ ^]^ is used to perform a 1 ns canonical REMD simulation with 12 replicas at 300–900 K, cf. Figure 1b (see further details in Section S1.3, Supporting Information).

Within AITD, the surface free energy *γ* of the sampled surface structures is determined as

(1) $$\begin{array}{ccc} \gamma (T,p) & = & \frac{G_{\mathrm{slab}}\left(T,p,N_{O},N_{\mathrm{Cu}}\right)-N_{\mathrm{Cu}}\mu_{{\mathrm{Cu}}_{\mathrm{bulk}}}\left(T,p\right)-N_{O}\left(\mu_{O}\left(T,p\right)\right)}{A_{\mathrm{surf}}}\\ & & -\;\gamma_{\mathrm{core}}\left(T,p\right), \end{array}$$

where *G*_slab_ is the energy of the surface slab containing a total of *N*_Cu_ Cu and *N*_O_ O atoms, *γ*_core_ is the frozen side's surface energy of the core slab, $\mu_{{\mathrm{Cu}}_{\mathrm{bulk}}}$ and $\mu_{O}=1/2\mu_{O_{2}}$ are the chemical potentials of bulk Cu metal and O_2_ gas, respectively, and *A*_surf_ is the surface area of the (7 × 7) surface unit‐cell. In SRS‐AITD, the (*T*, *p*)‐dependence for condensed matter (*G*_slab_ and $\mu_{{\mathrm{Cu}}_{\mathrm{bulk}}}$) is expected to cancel out in the difference in Equation (1) and is therefore neglected.^[^ ^22^, ^26^, ^44^ ^]^ For the *µ*_O_ = 1/2*E*_tot_(O_2_) + Δ*µ*_O_(*T*, *p*) gas‐phase chemical potential, the (*T*, *p*)‐dependence follows the ideal gas model^[^ ^13^, ^45^ ^]^ (see Section S1.5 for further details, Supporting Information).

To characterize the (disordered) surface oxidic structures, we classify the –O–Cu–O– ring and chain types present at the surface using a graph theory approach on the basis of the Networkx package,^[^ ^46^ ^]^ cf. Figure 1c. Analysis of the local atomic environments furthermore allows to automatically distinguish Cu bulk and Cu surface atoms coordinated by 0, 1, 2, and 3 oxygen atoms, CN_Cu‐O_ = 0 − 3, with further details provided in Section S1.4 (Supporting Information). All handling of atomic structures was performed by ASE and pymatgen.^[^ ^47^, ^48^ ^]^

## 3 Results and Discussion

### 3.1 Static Reductionist Sampling

**Figure 2:**
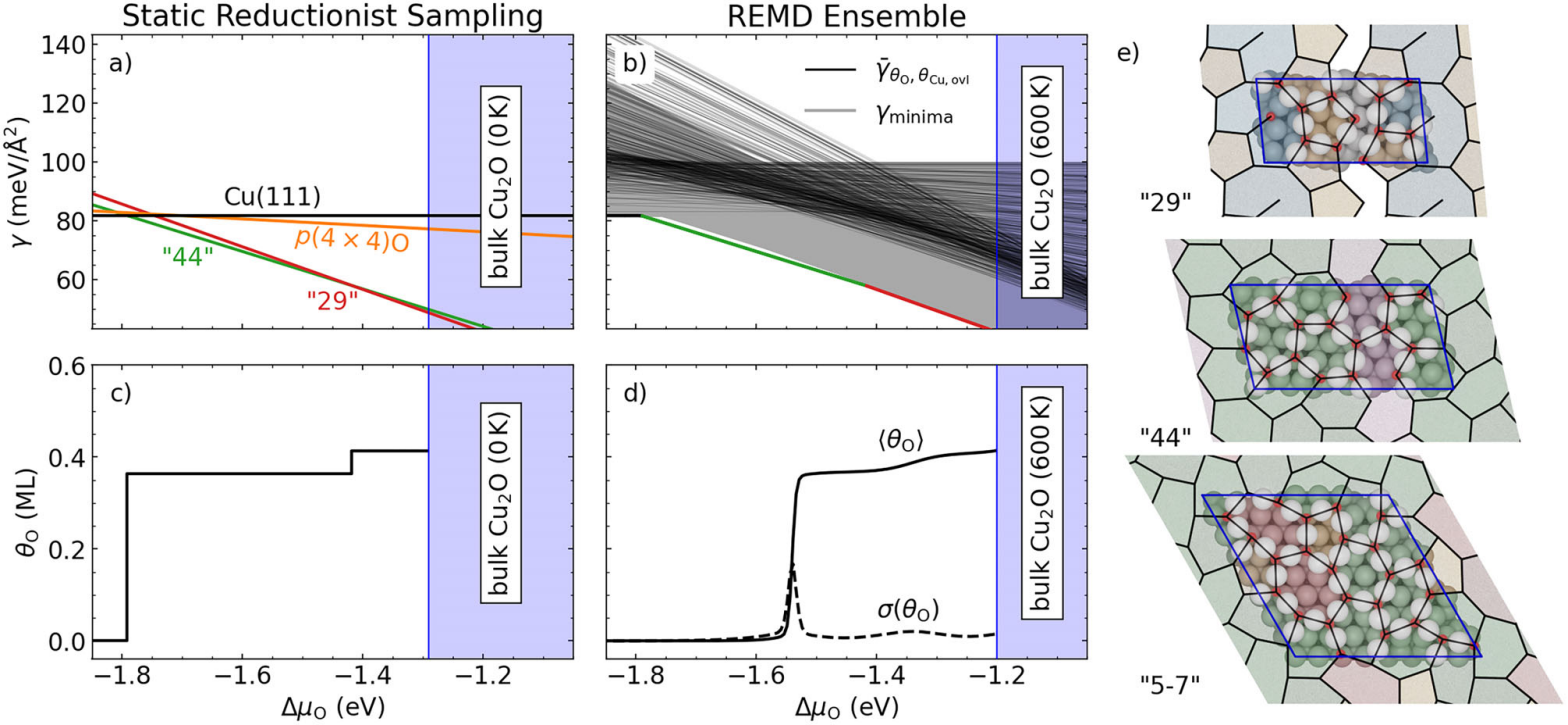
(Meta)stable structures and surface oxygen coverage *θ*_O_ during the initial oxidation of Cu(111). a–d) AITD surface free energy *γ* and concomitant *θ*_O_ as a function of the chemical potential Δ*µ*_O_ of the surrounding O gas‐phase and up to the thermodynamic stability range of bulk Cu_2_O. Contrasted are the results obtained with the prevalent static reductionist sampling (SRS) approach (a) and c)) based on a small pool of ordered candidate structures proposed in literature (see text for details) with those from REMD sampling (b) and d)). In (b), we display locally optimized structures sampled from the trajectories ($\gamma_{\mathrm{minima}}$, gray lines) and full thermodynamic averages over the different canonical REMD simulations at *T* = 600 K (${\bar{\gamma }}_{\theta_{O},\theta_{\mathrm{Cu},\mathrm{ovl}}}$, black lines). Boltzmann averaging the canonical averages smears out the unphysical discrete step‐function shape of *θ*_O_(Δ*µ*_O_) characteristic for the prevalent SRS. In b) the SRS‐AITD minimum energy curve from a) with coloring corresponding to the three stability regions (clean, “44”, “29”) is repeated for direct comparison, while d) also includes the standard deviation over the ensemble (dashed line). e) Top views of three prominent ordered surface oxide structures discussed in the literature:^[^ ^26^, ^28^ ^]^ “29”, “44”, and “5‐7” reconstructions, with Cu atoms in gray, and O atoms in red. The black lines visualize the interconnected –O–Cu–O– chain and ring network on the Cu(111) surface, which is extended beyond the periodic cell boundary (blue). The five‐, six‐, seven‐, nine‐, and ten‐member rings are colored orange, green, red, violet, and blue for better visualization.

The small pool of selected candidate structures considered in the prevalent SRS‐AITD approach is generally heavily inspired by chemical intuition and available experimental data. At late transition metal surfaces, this typically motivates consideration of suitable ordered superstructures of on‐surface chemisorbed O adsorbates at lowest coverages and various surface oxide reconstructions as observed in UHV studies. For the Cu(111) surface specifically, a wealth of such experimental work has firmly established that the initial oxidation is characterized by the formation of a roughened 2D network of –O–Cu–O– chains and rings.^[^ ^5^, ^7^, ^49^ ^]^ Depending on the details of the oxygen exposure, different periodic patterns emerge within this network. These include the frequently observed “29”, “44”, and “5‐7” structures depicted in **Figure** **2**e,^[^ ^22^, ^26^, ^28^ ^]^ as well as a metastable “8” structure^[^ ^50^ ^]^ and, more recently, a “41” structure.^[^ ^42^, ^51^ ^]^

The “44” and “29” configurations endure after prolonged annealing at high temperatures and have emerged as thermodynamically most stable phases in previous SRS‐AITD work.^[^ ^26^, ^28^ ^]^ Their names derive from their large unit‐cells, which correspond to 44 and 29 times the (1 × 1) Cu(111) surface unit‐cell, reshaped into complex $\sqrt{73}R5.8^{\circ }\;\times \;\sqrt{21}R\;-\;10.9^{\circ }$ and $\sqrt{13}R46.1^{\circ }\;\times \;7R21.8^{\circ }$ overlayer symmetries, respectively. We here consider the most recent “44” (*θ*_O_ = 0.36 ML, *θ*_Cu, ovl_ = 0.52 ML) and “29” (*θ*_O_ = 0.41 ML, *θ*_Cu, ovl_ = 0.55 ML) structural models as proposed by Zhu et al.,^[^ ^28^ ^]^ and refer to ref. [52] for a detailed survey on previously published models. Both ordered reconstructions feature –O–Cu–O– networks characterized by an intricate mixture of distorted rings, as depicted by the black lines in Figure 2e. Specifically, the “44” structure consists of six distorted six‐rings and a “peanut”‐shaped ten‐ring, while the “29” structure features three five‐rings, a “bean”‐shaped ten‐ring, and a continuous “zig‐zag” channel.^[^ ^28^ ^]^ The “5‐7” structure (*θ*_O_ = 0.38 ML, *θ*_Cu, ovl_ = 0.56 ML), slightly reduced and metastable to the “44” structure, is frequently observed after initial O_2_ dosage. It is named after its network patterns of four neighboring five‐ and seven‐rings (see Figure 2e). This pattern is hypothesized to reconstruct to four neighboring six‐rings, characteristic of the “44” structure, following further oxidation during extended annealing times.^[^ ^26^, ^28^ ^]^ At increasingly oxidizing conditions and metastable to the bulk oxidation to Cu_2_O,^[^ ^52^ ^]^ additional structures have been reported, including O‐decorated “44” and “29” superstructures and the rare “8” structure.^[^ ^7^, ^26^, ^28^ ^]^ In contrast to these –O–Cu–O– network structures, ordered superstructures of on‐surface chemisorbed adsorbates, like a suggested *p*(4 × 4)O motif, have never been directly observed at ambient conditions.^[^ ^22^ ^]^

In Figure 2a, we illustrate the SRS‐AITD approach for describing the initial surface oxidation of Cu(111) up to the thermodynamic Cu_2_O‐bulk oxidation, by using the “44”, “29”, “5‐7” and *p*(4 × 4)O as literature‐inspired pool of candidate structures. According to their differing O content, the surface free energies *γ* of these four structures (each computed in their respective surface unit‐cells) exhibit different gradients with respect to the oxygen chemical potential. As a result, the corresponding lines of the “44” and “29” reconstruction in Figure 2a intersect as Δ*µ*_O_ increases, confirming each structure as thermodynamically most stable in a distinct Δ*µ*_O_ region. In contrast, the *γ*‐lines of the “5‐7” and *p*(4 × 4)O remain at higher energies over the entire relevant chemical potential range up until the onset of bulk oxidation, again consistent with their empirically assigned metastability. Overall, SRS‐AITD thus provides a picture of the initial surface oxidation of Cu(111) that is in full agreement with the general consensus on experimental data.

However, this achievement sensitively depends on the structures considered in the candidate pool, which is an explicit human input and not an outcome of the modeling process. Furthermore, the “44” and “29” superstructures are identified as thermodynamically relevant in Figure 2a solely due to the specific models we considered. Older structural models in literature, that majorly consider much higher O coverages (*θ*_O_ > 0.5 ML), lead to much higher surface free energies and a corresponding incorrect classification as metastable, e.g., compared to the “5‐7” reconstruction.^[^ ^28^, ^52^ ^]^ The lack of any proper sampling of candidate structures in the SRS‐AITD approach also manifests itself in the non‐physical discontinuities of the predicted O coverage curve as a function of the O chemical potential (see Figure 2c). Since in SRS‐AITD, *θ*_O_(Δ*µ*_O_) is simply equated with the O content of the most stable structure, it exhibits discrete jumps at the predicted transitions from the clean to the “44” at Δ*µ*_O_ = −1.79 eV and from the “44” to the “29” phase at Δ*µ*_O_ = −1.42 eV.

### 3.2 Advanced Sampling

To address these limitations, we now leverage the MLIP‐enabled REMD sampling and replace the small hand‐selected pool of SRS candidate structures with an extensive set that systematically covers the configurational space. For this, we locally relax snapshots taken from the canonical REMD trajectories at 300 K in 2 ps intervals. Doing this for the 400 distinct trajectories for the Cu_*x*_O_*y*_ overlayer compositions that cover the entire range from the clean metal surface to a nearly complete double layer of epitaxial Cu_2_O(111), leads to a total of 180 000 structural models. As apparent from Figure 2b, the lowest energy configurations of this set come very close to the ordered reconstructions of the SRS pool, i.e., the sampling leads to structures of comparable stability in silico and without explicit experimental input as in the SRS‐AITD approach. Notable is only a small energetic gap of less than 3 meV/Å^2^ (≈3.3 meV/atom at the various compositions) to the “44” surface oxide that closes toward higher Δ*µ*_O_. This is a finite size effect, as the (7 × 7) surface unit‐cell employed in the sampling cannot commensurably accommodate the “44” and “29” structures. Instead, the sampling yields a multitude of defected and strained versions of these structures with similar geometric motifs that fit into the simulation cell, as we will further detail below. Less strain and defects are required for the smaller “29” reconstruction, rationalizing the reduction of the energetic gap to below 0.8 meV/Å^2^. Furthermore, the sampling identifies more than 2500 configurations that are within less than 2 meV/Å^2^ (≈2.2 meV/atom) to this ordered surface oxide and are thus energetically essentially degenerate.

This high degree of degeneracy reflects a low energetic penalty to rearrange the rings and chains in the polymeric –O–Cu–O– networks that form at the oxidizing surface. This is consistent with the significant heterogeneity and disorder of the surface reported experimentally and demonstrates that the ordered “44” and “29” reconstructions are just specific representations of this network that can be stabilized as larger domains after carefully tuned oxygen exposure, annealing and cooling. In situ and at intermediate temperatures, patches of these reconstructions will instead coexist with disordered regions exhibiting a wide variety of ring and chain motifs, as well as defects.

When aiming to capture this heterogeneity, observables like the O coverage can no longer simply be equated with those of the lowest‐energy configuration as done in SRS‐AITD. A straightforward alternative would be to Boltzmann average over the various low‐energy configurations of Figure 2b.^[^ ^53^ ^]^ In this, one would, however, neglect surface vibrational effects at the finite temperature. Such effects could be approximately introduced through harmonic corrections to the surface free energies of the individual locally‐optimized configurations.^[^ ^13^, ^54^, ^55^ ^]^ Notwithstanding, having the full canonical REMD simulations at hand allows us to address this more efficiently and accurately by directly obtaining the thermal surface free energy ${\bar{\gamma }}_{\theta_{O},\theta_{\mathrm{Cu},\mathrm{ovl}}}\left(\Delta \mu_{O},T\right)$ at a given temperature as a trajectory average,^[^ ^21^ ^]^ cf. Section S1.5 (Supporting Information) for details. Figure 2b includes the resulting lines for the 400 distinct compositions, over which we subsequently Boltzmann average to obtain the grand‐canonical expectation value for the O coverage as

(2) $$\left\langle \theta_{O}\left(\Delta \mu_{O},T\right)\right\rangle =\sum_{\theta_{O},\theta_{\mathrm{Cu},\mathrm{ovl}}}\theta_{O}\;\exp\left(\frac{-{\bar{\gamma }}_{\theta_{O},\theta_{\mathrm{Cu},\mathrm{ovl}}}\left(\Delta \mu_{O},T\right)A_{\mathrm{surf}}}{k_{B}T}\right)/Z,$$

with *k*_B_ the Boltzmann constant and the partition function

(3) $$Z\;=\;\sum_{\theta_{O},\theta_{\mathrm{Cu},\mathrm{ovl}}}\exp\left(\frac{-{\bar{\gamma }}_{\theta_{O},\theta_{\mathrm{Cu},\mathrm{ovl}}}\left(\Delta \mu_{O},T\right)A_{\mathrm{surf}}}{k_{B}T}\right)\;.$$

This average, as well as the simultaneously obtained standard deviation *σ*(*θ*_O_(Δ*µ*_O_, *T*)), contains all configurational and vibrational finite temperature effects, except an entropy contribution associated with particle insertion, i.e., the change in accessible phase space volume with the number of particles.^[^ ^56^, ^57^, ^58^, ^59^ ^]^

Figure 2d shows the resulting coverage curve and its standard deviation for an intermediate temperature of 600 K, representative of the annealing temperatures previously employed in experimental work.^[^ ^23^, ^25^, ^28^ ^]^ As expected, 〈*θ*_O_(Δ*µ*_O_, 600 *K*)〉 now exhibits smoothened phase transitions, and it still features two extended plateaus at similar coverages as the “44” and “29” reconstructions considered in SRS‐AITD, cf. Figure 2c. However, the transitions occur at significantly different O chemical potentials, with in particular the onset from the clean surface to the first plateau strongly upshifted from Δ*µ*_O_ = −1.79 eV by about 0.25 eV. A small part of this shift (0.03 eV) is simply due to the above mentioned finite size effect. Even in the (7 × 7) surface unit‐cell though, sampled optimized O‐containing structures (gray lines with negative slopes in Figure 2d) start to have lower surface free energies than the clean surface (horizontal line) above Δ*µ*_O_ = −1.76 eV. The dominant part of the onset shift is thus due to the finite temperature effects considered in the REMD‐based approach. Approximately correcting the *γ* of the optimized structures with vibrational contributions evaluated using the (surface and bulk) phonon band structure, cf. Section S1.5 (Supporting Information), reveals that vibrational effects make up for about half of the onset shift. Consistently, a corresponding Δ*µ*_O_ shift of about 0.1 eV is also seen for the onset of the stability region of the bulk oxide at 600 K in Figure 2d. The remaining shift of ∼0.1 eV can therefore be ascribed to entropic effects, in this case primarily the entropic stabilization of sparse O adsorbates or O‐decorated Cu adatom motifs. Such structures are significantly higher up in energy in the corresponding chemical potential range around ∼−1.7 eV, cf. the position of the *p*(4 × 4)O line in Figure 2a, but are sampled so frequently that they dominate the partition function at this intermediate temperature.

**Figure 3:**
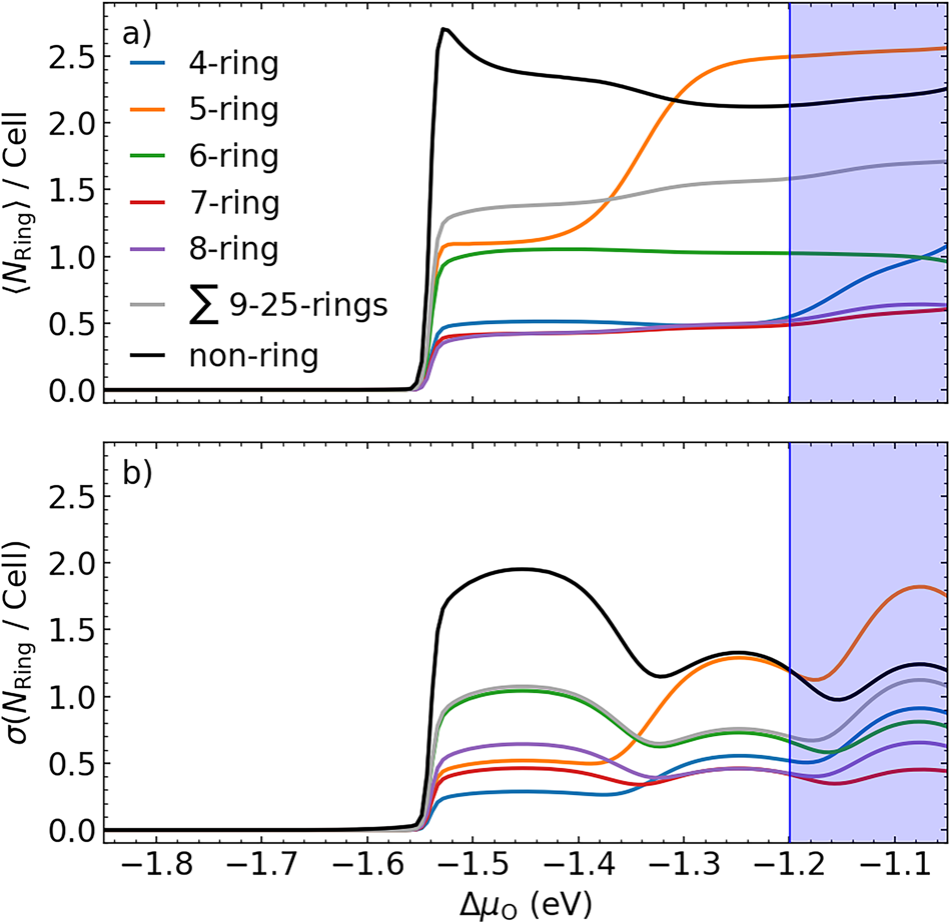
Statistics of –O–Cu–O– ring and chain types present on the oxidizing surface. a) Ensemble average 〈*N*_Ring_〉/Cell and b) standard deviation σ(*N*_Ring_/Cell) of motifs containing *N*_Ring_ Cu atoms in the simulation cell at 600 K. The purple area indicates the stability region of bulk Cu_2_O at this temperature. The various lines are extended into this region to indicate properties of the then metastable oxidized overlayer.

Numerous configurations also contribute to the partition function at higher Δ*µ*_O_. Even though the coverage curve exhibits two plateaus with similar coverages, the true state of the oxidized surface in this regime is thus more diverse than just the respective two ordered “44” and “29” reconstructions discussed in the SRS‐AITD context. We quantify the corresponding heterogeneity and disorder of the surface by evaluating the thermal average and standard deviation of the –O–Cu–O– ring and chain types present at the surface, as well as the O‐coordination of the surface Cu atoms, in an analogous way as done for the coverage before, cf. Equation (2) above. Indeed, as seen in the results compiled in **Figure** **3**, the standard deviation of the ring and chain motifs is mostly almost comparable to their actual average in the chemical potential range of the two plateaus. At the same time, with a very small *σ*(*θ*_O_), cf. Figure 2d, the O coverage is well defined. Together with a dominant one and twofold O‐coordination of Cu surface atoms (〈CN_Cu‐O_〉 = 1, 〈CN_Cu‐O_〉 = 2), cf. Figure S12 (Supporting Information), this data thus confirms the picture of the oxidized surface under these conditions as covered with a polymeric –O–Cu–O– network. This overlayer has a defined overall surface density, but high flexibility and variability in its network structure. As also reflected in the much smaller *σ*(*θ*_O_) peak in Figure 2d, the second phase transition to the 0.41 ML plateau at around Δ*µ*_O_ ≈ −1.35 eV is correspondingly also less pronounced than the first onset phase transition at around ‐1.55 eV. Essentially, the network is only a bit more densified to accommodate more O atoms at the surface. As apparent from Figure 3, this proceeds primarily through an increased compactification of remaining monomeric –O–Cu–O– units into five‐rings and larger (mostly channel‐like) ring structures. In an idealized way, this physics is also captured by the formal transition from the “44” to the “29” reconstruction, where prevailing six‐rings are replaced by five‐rings. In fact, also the abundance of five‐, six‐ and larger ring structures in the REMD ensemble is mirrored by the analog motifs exposed by these two ordered reconstructions. In contrast, we find a significant stabilization of four‐rings only at O chemical potentials that already fall into the thermodynamic stability region of bulk Cu_2_O, cf. Figure 3. Again, this is consistent with the observation of the metastable “8” structure containing such rings only after hyperthermal oxygen molecular beam synthesis.^[^ ^50^ ^]^

**Figure 4:**
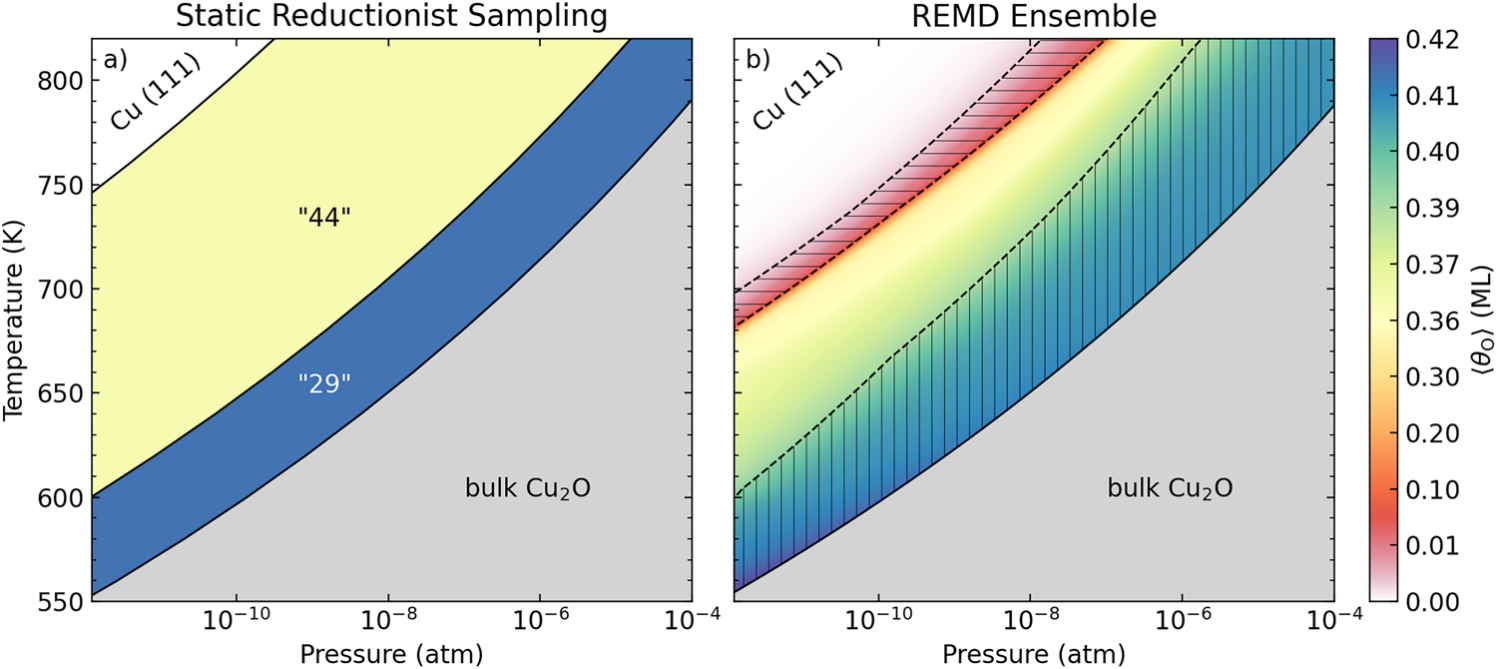
Ab initio thermodynamics (*T,p*) surface phase diagrams of the initial oxidation of Cu(111). a) Prediction based on the hand‐selected pool of experiment‐inspired ordered structures, see text for details. b) In silico prediction based on the REMD ensemble and including concomitant vibrational and configurational temperature effects. Apart from inducing a smoothening and in parts significant shifting of the phase transitions, this allows to identify the presence of additional species at the oxidized surface: O adatoms (horizontally hatched area) and highly oxidized Cu surface atoms (vertically hatched area). This presence is defined through the coverage of surface Cu atoms with one‐, two‐ and threefold O coordination number (CN_Cu‐O_= 1‐3): O adatoms (*θ*(CN_Cu‐O_ = 1) > 0.1 ML, and simultaneously *θ*(CN_Cu‐O_ = 1) > *θ*(CN_Cu‐O_ = 2), with the latter condition distinguishing from the formation of the polymeric –O–Cu–O– overlayer network) and highly oxidized Cu surface atoms (*θ*(CN_Cu‐O_ = 3) > 0.05 ML).

We summarize these insights in (*T*, *p*) surface phase diagrams in **Figure** **4**, where we contrast the according predictions from the prevalent SRS‐AITD approach and the present REMD sampling. In the prior case, the (*T*, *p*)‐dependence results from the trivial ideal‐gas type expansion of the 1D Δ*µ*_O_‐dependence shown in Figure 2a. We correspondingly obtain the previously discussed stabilization of the two ordered “44” and “29” surface oxide reconstructions and of bulk Cu_2_O at increasingly O‐rich environments, with infinitely sharp phase transitions following lines of constant O chemical potential. In the REMD‐based surface phase diagram, the *T*‐dependence derives instead from the explicit analysis of the REMD data in the shown temperature range, analogous to the above exemplarily discussed data at 600 K, cf. Section S1.5 (Supporting Information) for details. This smoothens and shifts the phase transitions, in particular the formation of the polymeric –O–Cu–O– network is delayed to almost four orders of magnitude higher oxygen pressure. We note that similarly sized deviations of SRS‐predicted phase boundaries from experimental findings in the ab initio thermodynamics literature have recurrently been ascribed to deficiencies of the employed DFT functional. Here, the shift results instead entirely from unaccounted vibrational and configurational contributions to the calculated SRS surface free energies. Despite this delayed onset, the polymeric network still prevails over a wide range of gas phase conditions. In this range, the surface state is quite heterogeneous and exhibits differing –O–Cu–O– chain and ring structures. This is fully consistent with the wealth of existing experimental characterization data, which even under carefully tuned oxygen exposures repeatedly reported the characteristic “44” and “29” structures to co‐exist in nanometer‐sized domains, while also bordering to strongly disordered regions of equal size.^[^ ^5^, ^7^, ^28^, ^42^, ^49^ ^]^

Beyond the –O–Cu–O– motifs, the appropriate thermal averaging over the REMD ensemble also reveals the presence of important further species at the oxidizing surface. Prominently, the surface phase diagram in Figure 4b exhibits an extended, entirely entropically stabilized region where the surface predominantly features isolated O adsorbates. Toward more O‐rich conditions, the sampled ensemble then also provides intriguing insight into potentially important minority species, an information completely absent in the simplistic SRS phase diagram. Toward the phase boundary signaling the formation of the polymeric –O–Cu–O– overlayer network, the O adsorption phase increasingly exhibits O‐stabilized Cu adatoms, an active site motif that has been suggested for several thermal^[^ ^30^, ^31^, ^32^ ^]^ and electrocatalytic^[^ ^60^, ^61^, ^62^, ^63^ ^]^ reactions. There is also a percent‐range presence of neither O nor Cu adatom coordinated Cu surface atoms even within the stability range of the disordered polymeric –O–Cu–O– overlayer (see Figure S12, Supporting Information) – a motif that is completely absent in the ordered “44” and “29” reconstructions. In even more O‐rich environments, but not yet in the stability region of the bulk oxide, we also find a very small presence of densely oxygen‐coordinated Cu atoms, comparable to Cu(II)O‐like environments. Significant amounts of this species have hitherto only been observed in harsh reaction conditions like plasma oxidation.^[^ ^64^ ^]^ Due to their distinct chemistry, all these motifs could make an important contribution to the overall surface functionality even if only present in marginal quantities, and it is the REMD sampling that allows to identify and quantify this presence.

## 4 Conclusion

Our work demonstrates how MLIP‐enabled advanced sampling allows to overcome well‐known deficiencies of the prevalent realization of ab initio thermodynamics in the study of surfaces in finite chemical environments. Prevalent cartoon‐style surface phase diagrams depend sensitively on a considered small pool of candidate structures and exhibit abrupt phase transitions. The sampling instead identifies the dominant phases and their stability ranges in silico, while appropriate thermal averaging over the sampled ensemble allows to capture vibrational and entropic effects. For the showcase of the initial oxidation of Cu(111), the consequences on the surface phase diagram are manifold and significant: O adatoms appear as a purely entropically stabilized new phase, phase transitions are shifted by orders of magnitude in pressure, and a more differentiated view emerges of the surface oxides that form over a wide range of environmental conditions. Previously focused ordered reconstructions are only select representations of a highly flexible and variable –O–Cu–O– polymeric network.

The sampled partition function provides a quantitative account of the various chains, rings and channels formed in this network, and thus provides a first atomic‐scale model for the heterogeneity and disorder frequently observed in experimental studies. This much more realistic picture of the oxidizing surface also extends to the prediction of minority species or minority surface geometric motifs. At the oxidized Cu(111) surface, this notably comprises O‐decorated Cu adatoms which have repeatedly been discussed as important active sites in a range of catalytic applications. While this underscores the new quality of the achieved first‐principles description, we also note that finite size effects in computationally tractable simulation cells remain. While they are mitigated by the flexibility of the polymeric network in the present case, this dictates further methodological work, to quantify and possibly correct these effects or to reach larger cells through further accelerated sampling.

## Conflict of Interest

The authors declare no conflict of interest.

## Supporting information

Supporting Information

## Data Availability

The code used for MLIP training and data analysis is available at https://github.com/Felixrccs/Cu‐111‐oxide.git. The MACE model as well as the corresponding MLIP training, test, and validation data, and local geometry optimized snapshots from the REMD simulations are available at https://doi.org/10.5281/zenodo.17088816.
