# Supplementary material for: Out of the Crystalline Comfort Zone: Sampling the Initial Oxide Formation At Cu(111)
Source: Adv Sci (Weinh). 2025 Oct 27;12(48):e13878. doi: 10.1002/advs.202513878 (PMC12752653; doi:10.1002/advs.202513878)
Supplement: Supplementary file 1 — Supporting Information [file ADVS-12-e13878-s001.pdf]

Supporting Information: Out of the Crystalline Comfort Zone: Sampling the Initial Oxide Formation at Cu(111)

Felix Riccius, Nicolas Bergmann, Hendrik H. Heenen,* and Karsten Reuter

Fritz-Haber-Institut der Max-Planck-Gesellschaft, Faradayweg 4-6, D-14195 Berlin, Germany

E-mail: heenen@fhi.mpg.de

Contents

1 Computational details **S-3**

1.1 DFT calculations S-3

1.1.1 Settings S-3

1.1.2 XC-functional S-3

1.2 MACE details S-5

1.2.1 MACE fit parameters S-5

1.2.2 Active learning procedure S-6

1.2.3 MLIP accuracy and validation S-9

1.3 Sampling simulations S-12

1.3.1 Sampling grid S-12

1.3.2 REMD S-13

1.3.3 Data collection from REMD simulations S-16

1.3.4 Geometry optimization S-16
1.4 Classification S-16
1.4.1 Clustering via coordination number S-16
1.4.2 Graph analysis S-17
1.5 *Ab initio* thermodynamics S-18
1.5.1 Static reductionist sampling S-19
1.5.2 Ensemble average S-20
1.5.3 O_2-gas and Cu-bulk reference chemical potentials S-21
1.5.4 Frozen surface's energy S-23
1.5.5 Vibrational contributions to the inner energy S-24

2 Structural data of sampled ensembles **S-26**
2.1 Characterization of the −O−Cu−O− network S-26
2.2 Metastable configurations S-27
2.3 Oxidation onset S-29
2.4 Formal oxidation state of surface Cu atoms S-30

References **S-33**

1 Computational details

1.1 DFT calculations

1.1.1 Settings

DFT calculations are performed using the Vienna Ab Initio Simulation Package (VASP 6.3.2).[S1,S2] We employ the projector-augmented wave (PAW) method[S3] in combination with a plane wave basis set under periodic boundary conditions and the Perdew-Burke-Ernzerhof (PBE) exchange-correlation (XC) functional.[S4] Furthermore, Gaussian smearing with $\sigma = 0.05$ is applied, and the electronic structure is converged within $1e^{-6}$ eV. We select an energy cutoff of 900 eV and a k-point density of $\texttt{KSPACING} = 0.11\text{Å}^{-1}$ which are conservative settings in comparison to previous work.[S5,S6] For the `KSPACING`-parameter we follow the VASP definition $N_i = \max(1, \text{ceiling}(|\mathbf{b}_i|2\pi/\texttt{KSPACING}))$, where N_i corresponds to the number of k-points, and b_i the reciprocal lattice vectors. For slab models, we sample only one k-point and apply a dipole correction perpendicular to the surface. DFT geometry optimizations were used in the generation of the initial training set (see Sec. 1.2.2), for which we employ VASP's internal conjugate gradient algorithm with a convergence criterion of $1e^{-5}$ eV.

1.1.2 XC-functional

Fig S1 depicts the bulk oxidation against the chemical potential of oxygen ($\Delta\mu_{\text{O}}$) as predicted by various XC-functionals and compared to the electrochemical bulk oxidation in aqueous conditions as measured in S7. We resorted to the latter, since we did not find precise data for the bulk phase transitions in the presence of O_2 gas.[S8] The Cu to Cu_2O phase transition is predicted at too high $\Delta\mu_{\text{O}}$ for all XC-functionals. For r2scan and PBSsol, the Cu_2O phase vanishes while it appears in a small $\Delta\mu_{\text{O}}$ window for PBE (red-shaded area) and in an even larger window for HSE06 (pink-shaded area). On the one hand, this behavior may be attributed to the inability of the employed XC-functionals, with the exception of HSE06, to accurately reproduce the antiferromagnetic spin state of CuO in spin-polarized DFT calcu-

lations, overestimating this phase's stability in comparison to Cu_2O. On the other hand, we compare geometry-optimized DFT-data (corresponding to 0K) to experimental data measured under standard conditions, and thus neglect any entropy contributions. Incorporating a vibrational free energy correction based on the harmonic approximation using phonopy,[S9] further broadens the O-chemical potential window where Cu_2O is thermodynamically favorable. Since we are solely interested in the Cu_2O monolayer formation and do not require an accurate description of the antiferromagnetic spin state of CuO, we choose the PBE XC-functional because it reproduces the phase stability in the correct order. Moreover, PBE has been previously employed for this system in literature.[S6,S10,S11]

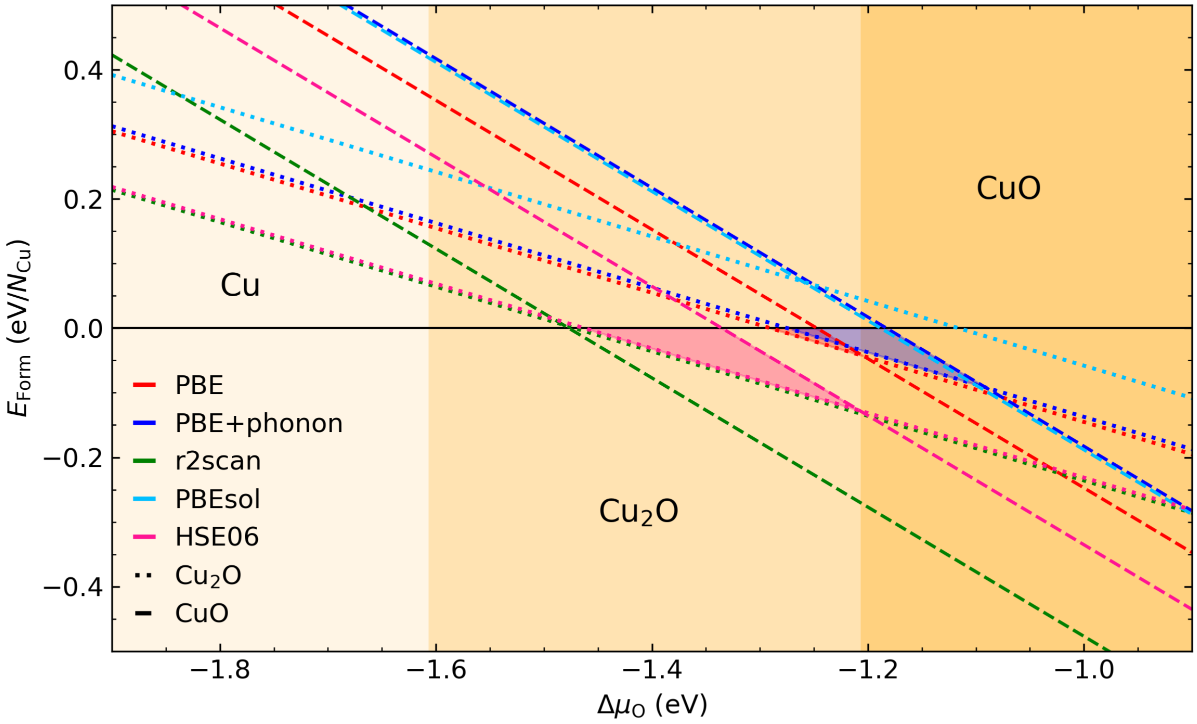

Figure S1: Comparison of bulk copper oxide phase transitions in respect to $\Delta\mu_O$. The graphs show the stability of bulk Cu(I)- and Cu(II)-oxides vs. Cu-metal as predicted via different DFT xc-functionals and the influence of phonons. The filled triangles visualize the potential range in which Cu(I)-oxide is the dominant phase. The light to dark orange background colors indicate the experimental phase transitions.[S7] The experimental transitions are retrieved from an aqueous system: E(Cu/Cu_2O) = -0.3 V, E(Cu_2O/Cu) = -0.1 V. To make the data comparable to our gas-phase reference, we convert the voltage to the O chemical potential of the oxygen atom in water at electrochemical standard conditions using the computational hydrogen electrode.[S12] To enable comparability to the gas-phase, we additionally switch the oxygen reference from water to the O_2 gas from Sec. 1.5.3

1.2 MACE details

1.2.1 MACE fit parameters

We use the following key parameters for our MACE fit: cutoff = 4.0 Å, number of interactions = 2, correlation = 3, max L = 3, maximum number of epochs = 1200, start swa epoch = 900. The combination of the cutoff and the number of interactions yields an effective cutoff of 8.0 Å. We deliberately choose a small cutoff as it ensures good computational performance

while still providing an accurate description of our condensed system.

1.2.2 Active learning procedure

Our machine-learned interatomic potential (MLIP) fitting starts from an initial reference training set, which is iteratively expanded within an active learning (AL) learning routine[S13–S15] (see Fig. S2). In each iteration of the AL-loop, the MACE potential is re-fitted based on the extended training set, which leads to a gradual improvement in both its accuracy and its description of the relevant chemical phase space until convergence with respect to the accuracy is observed. The applied AL scheme is fully automated and thus does not require human intervention during training. The resulting MLIP can accurately describe Cu and oxygen containing slab models (see Sec. 1.2.3). For future projects, we included hydrogen in the training set, enabling the description of hydroxyl groups and a possible transfer to aqueous systems. However, we exclusively focus on hydrogen-free systems in this work.

Initial training set

The initial training set includes three different types of structures: bulk models, dimers, and slab models. We include six bulk cells which included the as-available Material Project[S16] structures and its optimized variant, according to our DFT settings (see Sec. 1.1), of Cu (mp-30), Cu_2O (mp-361), CuO (mp-1692). We further include di-atomic structures (dimers) separated at a short distance to explicitly include short distances with acting repulsive forces, that help prevent atoms from collapsing into each other during the early training stages. These dimers include 3 $\times$ H-H (0.6-0.736 Å), 12 $\times$ O-O (0.85-1.91 Å), O-H (0.725 Å), 6 $\times$ Cu-Cu (1.3-2.037 Å), 4 $\times$ Cu-H (0.950-1.273 Å), 5 $\times$ Cu-O (1.075-1.562 Å). Finally, we include 49 slab models for Cu, Cu_2O, and CuO with the miller indices (100), (110), (111), (210), and (211). The slab models were created using pymatgen[S17] with a thickness of at least 5 atomic layers selecting only configurations that are symmetric in the z direction. The latter constraint leads to identical surface terminations within a single model. In addition, we

include all possible O- and Cu-terminated variants. These constraints are applied exclusively for the initial training set. Depending on the complexity of the slab and the underlying crystal system, the models contain between 20 and 176 atoms. For each slab model, we include the truncated bulk structure and its DFT-optimized structure (where the central layer(s) are fixed during optimization). 80 % of the slab models are randomly added to the training set and the remaining 20 % to the initial test set. Since the main objective of our MLIP is the description of slab models, dimers, and bulk structures are not contained within the test set.

AL-scheme

To rapidly build a comprehensive training set, we commenced the AL using a Gaussian Approximation Potential (GAP)[S18,S19] which can be trained significantly faster than neural network-based potentials such as MACE.[S20] During the later stages of the AL scheme, we change the MLIP to a MACE potential due to its superior performance and accuracy. Thus, the AL scheme shown in Fig. S2, differentiates between GAP- and MACE-based training. Once an MLIP fit is completed in the prior step, new structures are generated via Replica Exchange Molecular Dynamics (REMD) simulations[S21] of Cu, Cu_2O, and CuO slabs of varying surface orientation and oxygen content (see details Sec. 1.3.1 & 1.3.2). We bias the subsequent structure selection from the REMD trajectories, for the extension of the existing training set, to low-energy configurations by initially applying a Boltzmann-weighted pre-selection. In the GAP-based scheme, we then apply farthest point sampling (FPS)[S22] based on the SOAP descriptor and sample 10 configurations from the pre-selected configurations. In the case of the MACE-based scheme, we use a random selection and sample 20 configurations. We subsequently perform DFT calculations on the as-sampled configurations as well as their MLIP geometry-optimized structure (see Sec. 1.3.4). The inclusion of geometry-optimized and non geometry-optimized structures enables an accurate description of local minima as well as medium to high force configurations. 80 % of the sampled structures are added to the training set, and the remaining 20 % to the test set. Finally, a new MLIP is fitted and

the next iteration starts. The implementation of our AL scheme is based on the workflow package.[S23]

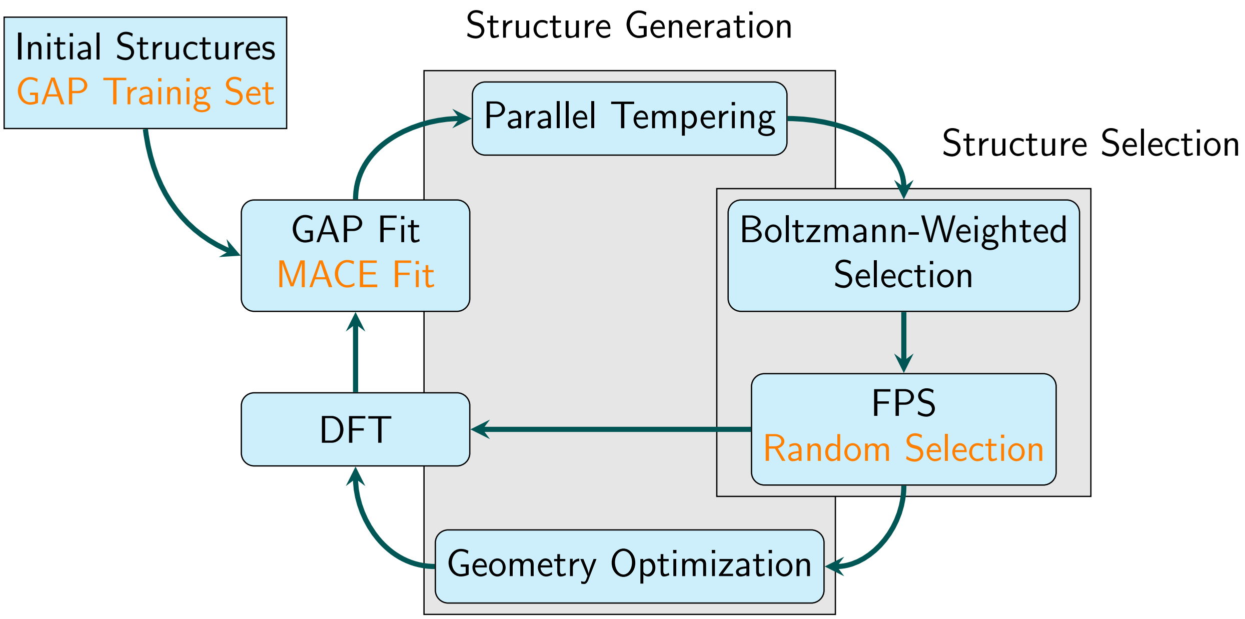

Figure S2: The active learning workflow: The black text visualize workflow with GAP as the MLIP, while the orange text shows the adaptations for MACE. The training starts from a GAP [MACE] fit based on the initial training set [final GAP training set]. REMD simulations and subsequent geometry optimizations are used to generate structures. A Boltzmann weighted selection combined with furthest point sampling (FPS) [a random selection] is used to select structures for DFT

The changes between the AL schemes originate in the different computational demands of the MLIPS: While a GAP fit is fast compared to a MACE fit (GAP-fit ≈ 1-2 h (CPU node); MACE-fit ≈ 9 h (A100 GPU)), the GAP-REMD simulation is slow compared to MACE (GAP-REMD ≈ 8 h (CPU node); MACE-REMD ≈ 0.5 h (A100 GPU)). Consequently, it is beneficial to minimize the number of MLIP fits for MACE- compared to GAP-based AL: In the GAP scheme, we perform a single REMD simulation per iteration, while ten parallel REMD simulations per iteration are executed in the MACE scheme. Although FPS has been commonly applied in GAP-based AL-scheme,[S15] MACE-based AL-schemes often sample based on uncertainty.[S24] However, uncertainty-based sampling requires multiple MACE-fits, as well as energy and force evaluations, and thus is related to a high computational cost.

Additionally, we do not require the same accuracy for high energy configurations, encountered at high temperatures ($T > 1000$ K), as compared to low energy configurations. That makes uncertainty-based sampling (which predominantly selects the former) not well suited for our application. Thus, we chose to exchange FPS through a random selection in the MACE scheme.

Boltzmann-weighted selection

Although the high temperature replicas from the REMD simulation are beneficial for an effective and fast phase space exploration, they mostly sample molten and high force structures. To bias our sampling towards medium- and low-force configurations that are relevant for our target phase space, we introduce a Boltzmann-weighted selection. The selection follows Eq. 1 and is applied to all data points drawn from a single REMD simulation. A structure i is accepted if a drawn random number is smaller than the probability $P(i)$, which depends on its relative energy $E(i)$. Instead of using a Boltzmann term ($k_\mathrm{B}T$), we multiply the maximum energy difference ($E_\mathrm{max} - E_\mathrm{min}$) by a factor of 0.2, where E_max is the highest, and E_min is the lowest energy contained within the data points that are drawn form all replicas originating from a single REMD simulation. This approach is robust and consistent with regard to the various cell sizes and chemical compositions we encounter during the AL scheme.

$$P(i) = \exp\left(-\frac{E(i) - E_\mathrm{min}}{0.2 \cdot (E_\mathrm{max} - E_\mathrm{min})}\right) \tag{1}$$

1.2.3 MLIP accuracy and validation

During and after training, we perform a vigorous validation of our MLIP to ensure that our potential can accurately describe our target system. The final MACE fit yields the DFT bulk lattice parameters of Cu, Cu_2O and CuO within 0.1 % (Tab. S1). The MLIP is able to accurately reproduce unseen DFT surface free energies with an accuracy < 1.8 meV/Å^2 (Tab. S2) and is almost quantitative in its descriptions of our surface system. The

learning curve during iterative training (Fig. S3) visualizes the increase in accuracy due to the change from GAP to MACE. At the end of the training, the MLIP fit yields a root mean square error (RMSE) of 2.89 meV/atom for energies, and $\mathrm{RMSE_{Forces}} = 148.0$ meV/Å on our test set. If high force structures ($F_{\mathrm{max}} < 3$ meV/Å) are excluded, one can observe even better performance on the remaining configurations ($\mathrm{RMSE_{Energy}} = 2.11$ meV/atom, $\mathrm{RMSE_{Forces}} = 86.0$ meV/Å). As shown in Fig. S3 our MACE potential yields a good force correlation with respect to the DFT forces. Furthermore, the mean force error for small forces – typical in structures near a minimum – is sufficiently low (with $f_{\mathrm{max}} \leq 30 - 50$ meV/Å) to enable local geometry optimizations (c.f. Fig. S3). Consequently, the MLIP reliably mirrors the contour of the DFT-based potential energy surface.

Table S1: Bulk predictions DFT vs MACE: Differences in total energy (ΔE) and lattice parameter (a) prediction.

Bulk composition	Δ $\mathrm{E_{DFT-MACE}}$ (meV/Atom)	Δa[%]
Cu	0.81	0.08
Cu_2O	-2.16	0.04
CuO	-0.67	0.00

Table S2: Surface predictions DFT vs MACE: Differences in total energy (ΔE) and surface free energy ($\Delta\gamma$). The compared slab models are the most dominant REMD sampled local minima, as well as slab models we prepared as suggested in literature.[S6,S11]

Slab model	Δ E (meV/Atom)	$\Delta\gamma$ (meV/Å^2)
$Cu_{22}O_{16}$@Cu-111	0.72	0.6
$Cu_{26}O_{18}$@Cu-111	0.84	0.72
$Cu_{28}O_{20}$@Cu-111	0.75	0.66
$Cu_{28}O_{22}$@Cu-111	0.59	0.52
Cu(111)	-0.82	-0.58
Lee "5-7"[S6]	0.85	0.74
Lee "44"+7O[S6]	-0.42	-0.37
Lee "29"+5O[S6]	0.61	0.56
Zhu "29"[S11]	1.63	1.41
Zhu "29"+1O[S11]	1.60	1.39
Zhu "44"[S11]	2.10	1.79
Zhu "44"+7O[S11]	0.71	0.81

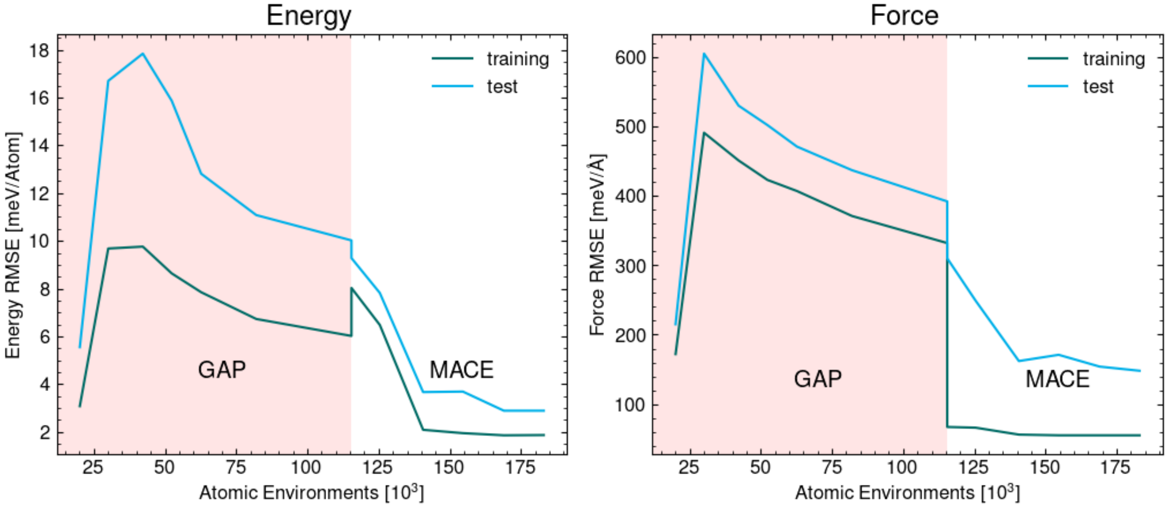

(a) Energy and force learning curve

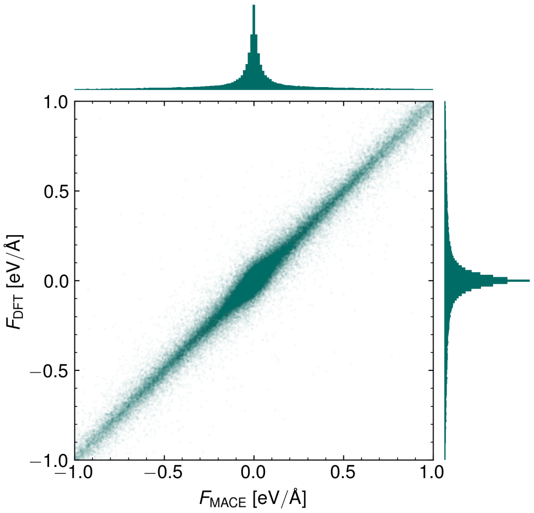

(b) Force correlation

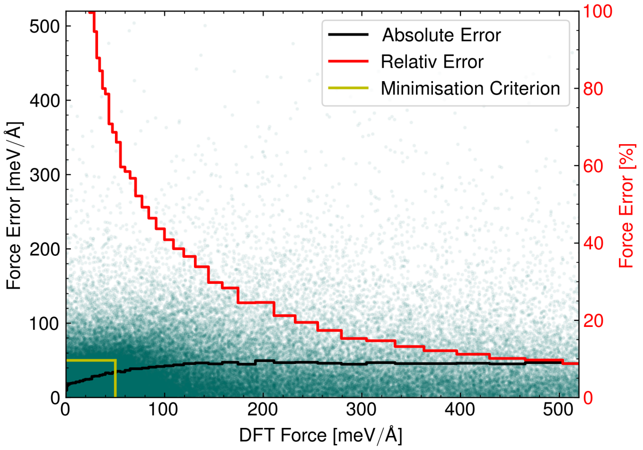

(c) Relative and absolut force error

Figure S3: Learning curves (a): on left-hand side the energy RMSE, on the right-hand side the force RMSE. The blue curves show the test set, the green curve the training set. The red background show the generations that are fitted with GAP and the white background the generations fitted with MACE. DFT-Force vs MACE-Force correlation plot (b): The histograms depict the DFT/MACE force distributions. Force error vs. DFT Force (c): The force components are displayed with regard to the force error. The black curve shows the absolute and the red curve shows the relative force error (each value is binned over 2000 data points). The yellow cube visualizes a force minimization criterion of 50 meV/Å.

1.3 Sampling simulations

1.3.1 Sampling grid

For our production runs, we use a 7×7 Cu(111) super cell, as it is the smallest commensurate cell with the $Cu_2O(111)$ surface.[S5] Our slab models contain a core of four Cu layers (Cu_{196} with a vacuum spacing of 25 Å), where the lowest layer is kept fixed during the simulation. We sample different degrees of oxidation grid-wise by adding 2 O atoms per oxidation state to the top surface of the core slab (additional $N_{\mathrm{O}} = 0, 2, 4, .., 30$). Furthermore, we vary the Cu atom content by adding Cu to the overlayer (additional $N_{\mathrm{Cu,ovl}} = 0, 2, 4, .., 48$), in order to enable reconstruction of the clean Cu(111) surface into a realistic Cu−O monolayer (ML). The 16 possible N_{O}, and the 25 $N_{\mathrm{Cu,ovl}}$ concentrations result in a composition grid containing 400 distinct combinations, covering the relevant phase space. The initial configurations are generated randomly as visualized in Fig. S4.

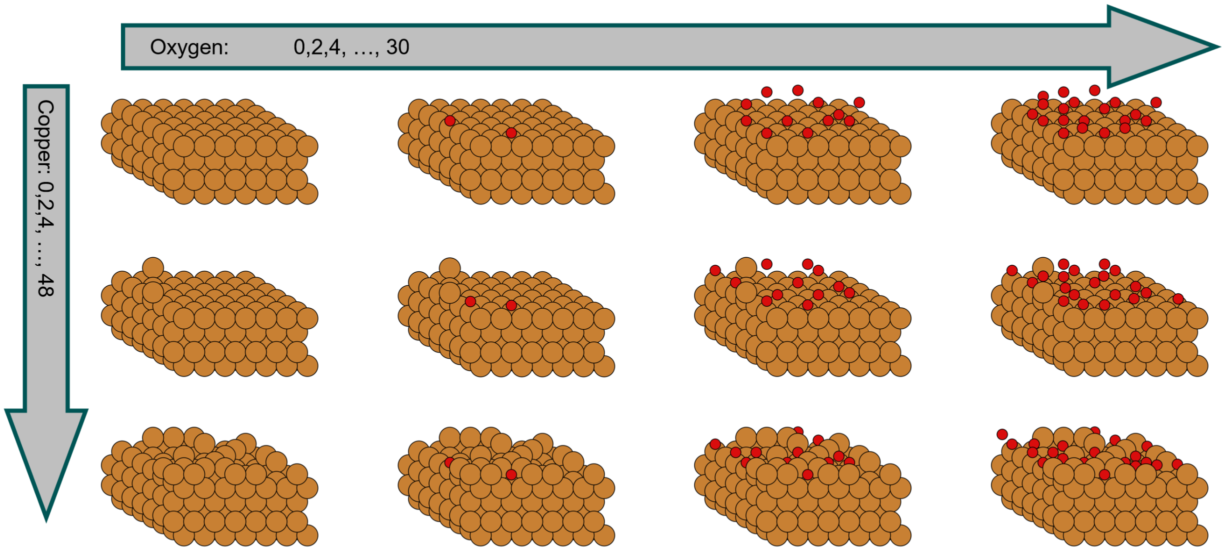

Figure S4: Exemplary overview of slabs-models used to initialize the REMD simulations, spanning the sampled composition space. The generation of the configurations is randomized and automated.

During MLIP training, we use slab models for Cu, Cu_2O, and CuO with various surface terminations ((100), (110), (111), (210), and (211)) as basis for the initial REMD configura-

tions. For slab models where a stoichiometric surface termination is not realizable we include both the O rich and poor variation, leading in total to 19 different basis slab-models. The slab models include at least five layers and the supercell extends between 7.7 and 14.9 Å(vs. 18.0 Åin the target 7×7-cell) along the bulk directions. The latter is chosen to ensure (in the majority of cases) that the smallest lattice parameter is greater than twice the GAP cutoff in order to avoid inclusion of symmetric environments in the SOAP descriptors through the periodic boundary conditions. This design leads to slab models containing between 50 and 200 atoms (compared to $\geq$ 196 atoms in the target 7×7-cell). To capture a more diverse chemistry, we modify the 19 basis slab models by randomly adding O atoms in the case of Cu-metal and removing O atoms in the case of Cu_xO systems and adding H atoms in both cases. The amount of O atoms we add / remove is randomly determined, and the base slab randomly selected each REMD simulation. This procedure results in 110 different initial configurations that include all 19 models with an equal proportion, within the AL-training scheme.

1.3.2 REMD

We perform REMD simulation using the LAMMPS code and its REPLICA package. We run trajectories for 1 ns via Nose-Hoover style time integration with a 100 fs damping parameter and a 1 fs time step. Our REMD simulations contain 12 replicas with temperatures according to the ratio $T_{n+1}/T_n \approx 1.11$ for ideal replica exchange (from which follows T = 300, 332, 366, 405, 447, 494, 546, 604, 667, 737, 814, 900 K). This temperature setup leads to an optimal swap acceptance ratio of 20 %,[S21] and allows efficient parallelization over four GPUs. In addition, we use a swap-attempt rate of 2 ps, which is larger than the energy auto-covariance time[S25] (see Fig. S5), thus preventing multiple back and forth swaps between adjacent replica. For some of the high O coverage configurations ($N_O >= 20$) O_2 evolution and subsequent desorption is observed during the simulation's equilibration (first 5 ps). To avoid O_2 evolution during equilibration, we start the REMD simulation from a different

initial structure: Instead of deriving the initial configuration from a clean Cu(111) surface, we add O atoms to a snapshot from a successful REMD simulation with a lower O coverage.

For the MLIP training we are only interested in phase space exploration and not in thermodynamically consistent ensembles. Thus, we use a modified, more efficient REMD setting within the AL-scheme, with only 7 replicas (T= 600, 708, 835, 986, 1163, 1373, 1620 K), which we run for 50 ps, and attempt a swap every 1 ps. Additionally, we use an adapted REMD implementation: The Boltzmann criterion is based on a normalized energy ($E_{\text{norm}} = E_{\text{tot}} * 10/N_{\text{Atoms}}$) instead of the total energy (E_{tot}), which leads to an increased swapping probability, enabling REMD simulations with fewer temperatures/replicas.

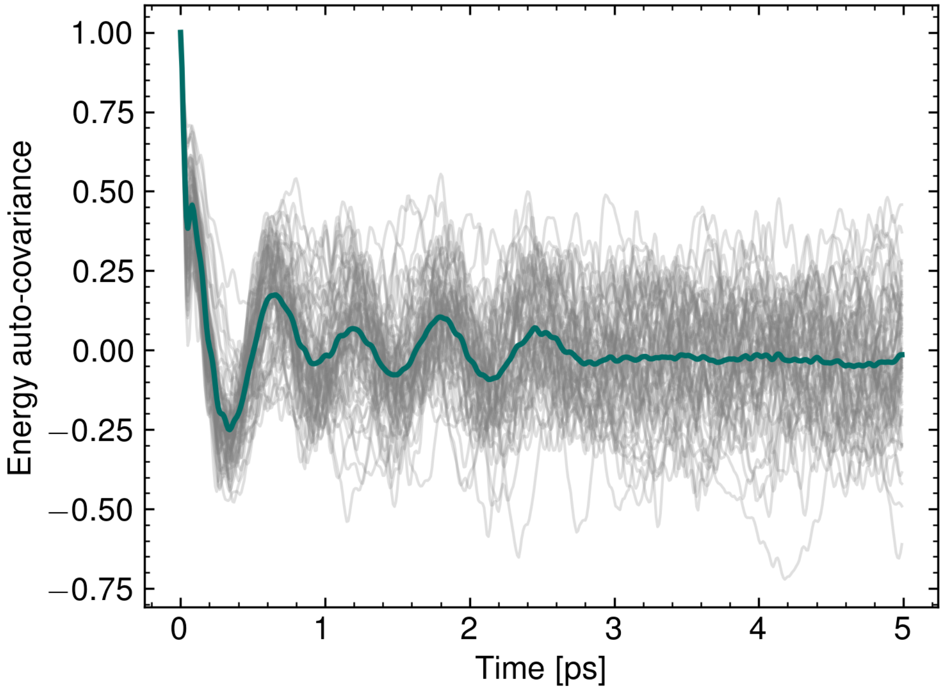

Figure S5: Energy auto-covariance of a Cu_2O ML on Cu-slab. We calculate the energy auto-covariance function $K_{EE}(\tau)$ by extracting uninterrupted trajectories (without a swap), with a minimum length of 10 ps from all replicas of a single REMD simulation. We computed the Energy auto-covariance K_{EE} in dependence of the lag-time τ via $K_{EE}(\tau) = \sum_{t=0}^{N} [E(t) - < E >] [E(t+\tau) - < E >]$ with E(t) as the energy at time t, $\langle E \rangle >$ the mean energy in the considered interval, N = 4 ps (400 steps), $\tau_{\text{max}} = 5$ ps (500 steps).

We calculate the running average of the REMD simulations to analyze the convergence

behavior. As shown exemplarily in Fig. S6, the individual replicas exhibit a clear energy convergence. To quantify the reminiscent energy drift, we perform a linear regression on the last 100 ps (last 22 %) of the running average. We find that the mean absolute slope for all REMD simulations is 0.39 meV/ps, with a maximum slope of 2.49 meV/ps and a minimum slope of -2.75 meV/ps). These values are an order of magnitude smaller than those reported for AIMD simulations,[S26] thus we assume that the REMD simulations are converged.

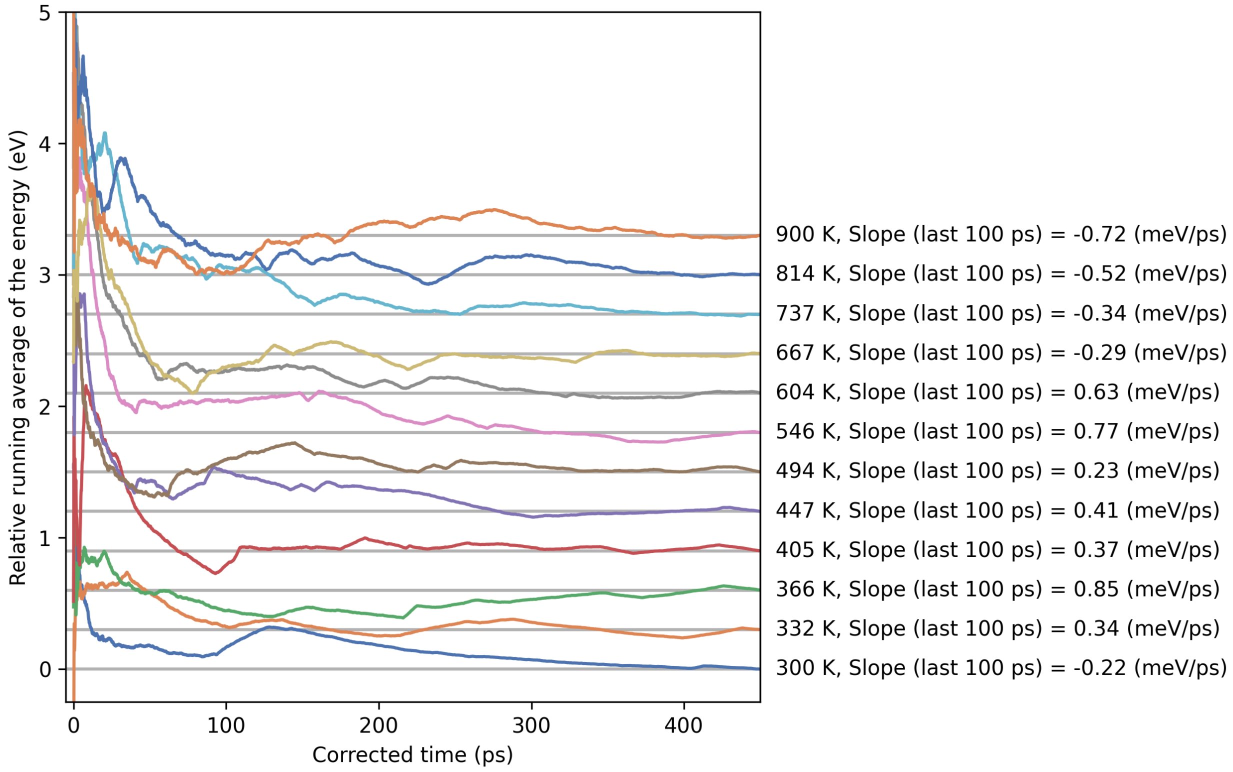

Figure S6: The running averages of the relative energy vs the corrected time shown for various replica of an exemplary REMD simulation for Cu26O18@Cu111. The horizontal gray lines show the mean relative energy, which is set to zero for the 300 K replica and plotted with an increasing offset for the replicas of the increasing temperatures. The corrected time omits the first 100 ps as well as 1 ps after each swap consistent with our data acquisition (Sec. 1.3.3). We further perform a linear regression over the last 100 ps of the running average to analyze the slope, indicative of the reminiscent energy drift, which is annotated on the right of the corresponding running average.

1.3.3 Data collection from REMD simulations

For evaluation of i.e. structural properties from the REMD simulations (see. Sec. 1.5.2), we need to extract uncorrelated snapshots from the canonical ensembles. To ensure that the snapshots are uncorrelated, the time between two adjacent snapshots should be larger than the energy auto-covariance time (c.f. Fig. S5). Thus, from all recorded snapshots (every 100 fs), we do a sub-selection so that all snapshots are at least 1 ps after a swap attempt and other selected snapshots. In addition, the data collection is commenced after an equilibration time of 100 ps. This leads to a fixed sample size of 450 snapshots per canonical ensemble (composition/temperature).

To determine the energy averages of the canonical ensembles (see Eq. 6), we use every recorded data point 1 ps after a swap attempt and 100 ps after the initialization of the simulation. We save the energies every 10 fs, thus accumulating 45,000 data points per canonical ensemble (i.e. per temperature/composition).

1.3.4 Geometry optimization

We use our MACE potential to perform local geometry optimizations with the ASE preconditioned linear BFGS implementation (PreconLBFGS). The force convergence threshold is set to 50 meV/Å.

1.4 Classification

We analyze the −O−Cu−O− network forming on the Cu(111) surface and investigate local Cu environments, aiming to obtain a more detailed picture of the surface configurations and their structural composition.

1.4.1 Clustering via coordination number

We classify Cu atoms according to their bulk character and O-coordination number (CN) of Cu. Instead of using a hard cutoff criterion, which would lead to coarse classification due

to structural fluctuations, we classify on basis of the MACE descriptor[S20] which is a smooth representation of our local Cu environments. To this end, we employ the workflow of the DECAF package,[S27] where the complex MACE descriptor is reduced in its dimensionality and consecutively clustering is applied. For the first step we use a principal component analysis (PCA) on 280.000 environments, a manageable subset of the descriptors drawn from all REMD simulations that are part of the production run. From the first two components, we extract four clusters via Hierarchical Density-Based Spatial Clustering of Applications with Noise (HDBscan)[S28] with a minimum cluster and sample size as 7000 and one, respectively. The four distinct clusters follow as Cu-bulk-like environments and three differently O-coordinated Cu-surface environments which show within a radius of ≈ 2.2 Å $CN_{Cu-O}= 0$, $CN_{Cu-O}= 1$, and $CN_{Cu-O} \geq 2$. This classification is also used to color-code the Cu atoms in rendered atomic structures.

1.4.2 Graph analysis

We apply a graph-based analysis to determine the size and quantity of rings that form in the −O−Cu−O− network. The Networkx package[S29] is used to create a graph network where the O atoms are defined as nodes. Two nodes are connected by an edge if a Cu atom is located between two nodes with a distance to both nodes smaller than 2.2 Å. We retrieve a list of all possible rings with a size smaller than 26 from the graph. This list includes redundant ringed structures, e.g. chains traversing the periodic cell or large rings that are superpositions of smaller rings. We sort out these invalid rings, by looping over all possible rings in ascending ring size, and evaluating the three following criteria (see Fig. S7): First, we check whether a ring ends in the same periodic cell where it originated. Second, we remove all edges from our network that are already part of two rings and consequently can not be part of any larger ring. Third, we map the ring back to real-space and evaluate whether any smaller ring is located within the ring's boundaries.

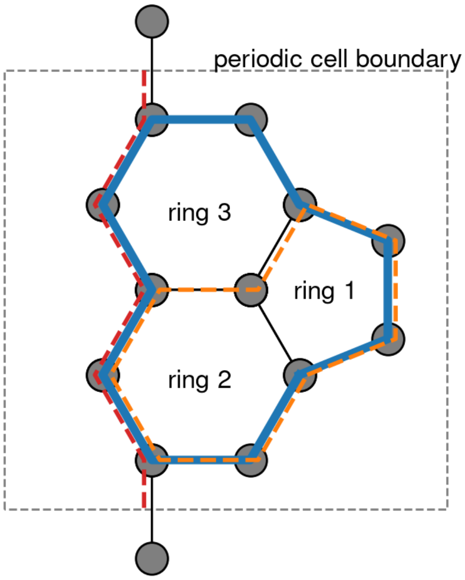

Figure S7: Schematic of an exemplary 2D-ring network under periodic boundary conditions. The nodes are visualized as gray dots which are connected by edges (lines). The shown network consists of a single 5-ring (ring 1) and two 6-rings (rings 2 and 3). The colored paths visualize invalid rings returned from the graph. Red visualizes a chain traversing the periodic cell, blue and orange are superpositions of smaller rings.

1.5 *Ab initio* thermodynamics

In all *ab initio* thermodynamics (AITD) approaches followed in this work, the surface free energy γ of the Cu(111)-oxide surface models is computed from the Gibbs free formation energy G_f of the interface between bulk Cu and O_2 gas.[S30] The surface energy is calculated following the basic formula:

$$\begin{aligned}\gamma(T,p) &= \frac{G_\mathrm{f}}{A_\mathrm{surface}} - \gamma_\mathrm{core}(T,p) \\ &= \frac{G_\mathrm{slab}(T,p,N_\mathrm{O},N_\mathrm{Cu}) - N_\mathrm{Cu}\mu_{\mathrm{Cu}_\mathrm{bulk}}(T,p) - N_\mathrm{O}(\mu_\mathrm{O}(T,p))}{A_\mathrm{surface}} - \gamma_\mathrm{core}(T,p)\end{aligned} \tag{2}$$

where G_slab is the Gibbs free energy of the slab model containing N_Cu Cu and N_O O atoms, γ_core is the frozen side's surface energy of our non-symmetric slab (see details in Sec. 1.5.4). $\mu_{\mathrm{Cu}_\mathrm{bulk}}$ as well as $\mu_\mathrm{O} = 1/2\mu_{\mathrm{O}_2}$ correspond to the chemical potentials referenced to Cu bulk and O_2 gas, respectively, and A_surface is the surface area of the simulation cell. The

dependence of the energies on temperature T and pressure p varies for the individual terms of the numerator in Eq. 2 and with respect to the approximations of the different sampling approaches that we elaborate in the following.

1.5.1 Static reductionist sampling

In the static reductionist sampling (SRS) approach, *ab initio* thermodynamics are combined with a sparse pool of structures, which are usually sampled with DFT based on human intuition, often guided by experimental data.[S6,S30,S31] This approach utilizes the assumption that the influence of temperature and pressure on condensed matter (i.e. G_{slab} and μ_{Cu}) is negligible at low temperatures. Therefore, the system can be described by local minima configurations and their corresponding potential energy E_{slab}. This leads to a simplification of Eq. 2:

$$\gamma(T,p) = \frac{E_{\mathrm{slab}}(N_{\mathrm{O}}, N_{\mathrm{Cu}}) - N_{\mathrm{Cu}}\mu_{\mathrm{Cu_{bulk}}} - N_{\mathrm{O}}\mu_{\mathrm{O_{gas}}}(T,p)}{A_{\mathrm{surface}}} - \gamma_{\mathrm{core}} \tag{3}$$

where the only T and p dependence remains in O chemical potential. We can substitute $\mu_{\mathrm{O_{gas}}}(T,p) = \mu_{\mathrm{O_{gas}}}(0\ \mathrm{K}, p_0) + \Delta\mu_{\mathrm{O}}$ and therefore express Eq. 3 in dependence of $\Delta\mu_{\mathrm{O}}$:

$$\gamma(\Delta\mu_{\mathrm{O}}) = \frac{E_{\mathrm{slab}}(N_{\mathrm{O}}, N_{\mathrm{Cu}}) - N_{\mathrm{Cu}}\mu_{\mathrm{Cu_{bulk}}} - N_{\mathrm{O}}(\mu_{\mathrm{O_{gas}}}(0\ \mathrm{K}, p_0) + \Delta\mu_{\mathrm{O}})}{A_{\mathrm{surface}}} - \gamma_{\mathrm{core}} \tag{4}$$

Furthermore, one assumes that the configuration that is lowest in energy is solely determining the phase. This results in the following probability of finding the configuration i on the surface:

$$P_i(\Delta\mu_{\mathrm{O}}) = \begin{cases} 1, & \text{if } \gamma_i(\Delta\mu_{\mathrm{O}}) = \min(\boldsymbol{\gamma}(\Delta\mu_{\mathrm{O}})) \\ 0, & \text{if } \gamma_i(\Delta\mu_{\mathrm{O}}) > \min(\boldsymbol{\gamma}(\Delta\mu_{\mathrm{O}})) \end{cases}, \tag{5}$$

where $\boldsymbol{\gamma}(\Delta\mu_{\mathrm{O}}) = [\gamma_0(\Delta\mu_{\mathrm{O}}), \gamma_1(\Delta\mu_{\mathrm{O}}), ..., \gamma_{N_{\mathrm{total}}}(\Delta\mu_{\mathrm{O}})]$.

1.5.2 Ensemble average

In this approach, we discard some of the previous approximations made in Eq. 3 and retrieve surface free energies from the REMD trajectories. From the $N_{\mathrm{snapshots}}$ that compose a canonical ensemble drawn from an REMD simulation with a given temperature T, as well as the O and Cu over-layer coverages θ_{O} and $\theta_{\mathrm{Cu,ovl}}$, we determine the energy average as

$$\bar{E}_{\theta_{\mathrm{O}},\theta_{\mathrm{Cu,ovl}}}(T) = \frac{1}{N_{\mathrm{snapshots}}} \sum_{i}^{N_{\mathrm{snapshots}}} E_i \quad , \tag{6}$$

where $\bar{E}_i$ corresponds to the total (potential + kinetic) energy of the individual snapshots. Even though our condensed phase free energies include the temperature T, we still neglect the p dependence. As in the previous approach, p is included in $\Delta\mu_{\mathrm{O}}$ for the O_2 gas phase reference. This leads to a simplification of Eq. 3 and an expression for the thermal surface free energies:

$$\bar{\gamma}_{\theta_{\mathrm{O}},\theta_{\mathrm{Cu,ovl}}}(T, \Delta\mu_{\mathrm{O}}) = \frac{\bar{E}_{\theta_{\mathrm{O}},\theta_{\mathrm{Cu,ovl}}}(T) - N_{\mathrm{Cu}}\bar{\mu}_{\mathrm{Cu_{bulk}}}(T) - N_{\mathrm{O}}(\mu_{\mathrm{O_{gas}}}(0\ \mathrm{K}, p_0) + \Delta\mu_{\mathrm{O}})}{A_{\mathrm{surface}}} - \bar{\gamma}_{\mathrm{core}}(T) \quad , \tag{7}$$

where $\bar{\mu}_{\mathrm{Cu_{bulk}}}(T)$ is also retrieved from reference MD simulations (see Sec. 1.5.3). The canonical ensembles span a uniform subset of the corresponding grand canonical ensemble, thus allowing the formulation of a generalized partition function $\mathcal{Z}$ based on canonical ensembles:

$$\mathcal{Z} = \sum_{\theta_{\mathrm{O}},\theta_{\mathrm{Cu,ovl}}} \exp\left(\frac{-\bar{\gamma}_{\theta_{\mathrm{O}},\theta_{\mathrm{Cu,ovl}}}(\Delta\mu_{\mathrm{O}}, T) A_{\mathrm{surf}}}{k_{\mathrm{B}}T}\right) \quad , \tag{8}$$

where k_B is the Boltzmann constant. This allows the determination of probabilities and consequently expectation values for our system:

$$\begin{aligned}\langle A(\Delta\mu_\mathrm{O},T)\rangle &= \sum_{\theta_\mathrm{O},\theta_\mathrm{Cu,ovl}} \langle A_{\theta_\mathrm{O},\theta_\mathrm{Cu,ovl}}\rangle \exp\left(\frac{-\bar{\gamma}_{\theta_\mathrm{O},\theta_\mathrm{Cu,ovl}}(\Delta\mu_\mathrm{O},T)A_\mathrm{surf}}{k_\mathrm{B}T}\right)/\mathcal{Z} \\ &= \sum_{\theta_\mathrm{O},\theta_\mathrm{Cu,ovl}} \langle A_{\theta_\mathrm{O},\theta_\mathrm{Cu,ovl}}\rangle p_{\theta_\mathrm{O},\theta_\mathrm{Cu,ovl}}(\Delta\mu_\mathrm{O},T) \quad ,\end{aligned} \tag{9}$$

where $\langle A_{\theta_\mathrm{O},\theta_\mathrm{Cu,ovl}}\rangle$ is the expectation value of A in the canonical ensemble with θ_O and $\theta_\mathrm{Cu,ovl}$ and $p_{\theta_\mathrm{O},\theta_\mathrm{Cu,ovl}}(\Delta\mu_\mathrm{O},T)$ the corresponding probability. Additionally we can evaluate the standard deviation of A. In cases where $\langle A_{\theta_\mathrm{O},\theta_\mathrm{Cu,ovl}}\rangle$ has zero standard deviation (i.e. the oxygen coverage), we determine σ_A as follows:

$$\sigma_A = \sqrt{\sum_{\theta_\mathrm{O},\theta_\mathrm{Cu,ovl}} p_{\theta_\mathrm{O},\theta_\mathrm{Cu,ovl}}(\Delta\mu_\mathrm{O},T)\left(\langle A_{\theta_\mathrm{O},\theta_\mathrm{Cu,ovl}}\rangle - \langle A(\Delta\mu_\mathrm{O},T)\rangle\right)^2} \tag{10}$$

If $\langle A_{\theta_\mathrm{O},\theta_\mathrm{Cu,ovl}}\rangle$ has a nonzero standard deviation (i.e. ring distributions), we use error propagation to calculate the overall variance of A:

$$\sigma_A = \sqrt{\sum_{\theta_\mathrm{O},\theta_\mathrm{Cu,ovl}} p_{\theta_\mathrm{O},\theta_\mathrm{Cu,ovl}}(\Delta\mu_\mathrm{O},T)^2\sigma^2_{A,\theta_\mathrm{O},\theta_\mathrm{Cu,ovl}}} \tag{11}$$

1.5.3 O_2-gas and Cu-bulk reference chemical potentials

Our oxygen reference is based on a O_2 molecule in vacuum (spin-polarized DFT, $E_{\mathrm{O}_2} = -9.874$ eV). Additionally, we add the Zero-point correction ($E_\mathrm{ZPE} = 0.096$ eV), which is determined via the ASE Thermochemistry module. This yields a 0 K reference of $\mu_\mathrm{O}(0\ \mathrm{K}, p_0) = -4.889$ eV. The temperature and pressure adjustment is done via the ideal gas approximation:[S31]

$$\mu_\mathrm{O}(T,p) = \mu_\mathrm{O}(0\ \mathrm{K},p_0) + \Delta\mu_\mathrm{O}(T,p_0) + \frac{1}{2}k_\mathrm{B}T\ln\left(\frac{p}{p_0}\right) \tag{12}$$

where the temperature dependence $\Delta\mu_{\mathrm{O}}(T, p_0)$ is drawn from NIST thermochemical tables.[S32]

The Cu bulk reference changes for the different approaches. For the static approaches, we obtain our bulk chemical potentials from the geometry-optimized bulk cell (0 K row in Tab. S3). Following literature,[S6,S30] we assume the vibrational, configuration, and pV-contribution to be negligible for low temperatures, and we can define:

$$\mu_{\mathrm{Cu}}(T, p) \approx \mu_{\mathrm{Cu}}(0\ \mathrm{K}) = \frac{G_{\mathrm{Cu}}(0\ \mathrm{K})}{N_{\mathrm{Cu}}} \tag{13}$$

where $G_{\mathrm{Cu}}(0\ \mathrm{K})$ is the potential energy of the geometry optimized bulk cell and N_{Cu} the number of the herein contained Cu atoms.

In the case of the ensemble average approach, we retrieve our chemical potentials from NPT simulations ($p = p_0$):

$$\bar{E}(T) = \frac{1}{N_{\mathrm{snapshots}}} \sum_{i}^{N_{\mathrm{snapshots}}} E_i(T) \tag{14}$$

where E_i are the energies of N_{samples} retrieved from the NPT ensemble. This leads to temperature dependence on chemical potentials:

$$\mu_{\mathrm{Cu}}(T, p) \approx \bar{\mu}_{\mathrm{Cu}}(T) = \frac{\bar{E}_{\mathrm{Cu}}(T)}{N_{\mathrm{Cu}}} \tag{15}$$

The chemical potential retrieved from the NPT ensemble leads to inconsistency with our slab models, where the surface area and shape are fixed and the model is thus prohibited from thermal expansion perpendicular to the surface plane. This inconsistency presents a constant offset and does not affect relative energies that are more important for our study. Nevertheless, we set out to quantify it by evaluating an alternative chemical potential from the bulk region of a slab model. The difference in the bulk and the slab-derived chemical potential within the temperature range of interest is small enough to be negligible ($\Delta\mu_{\mathrm{Cu}} < 3$

meV, $\Delta\gamma < 0.5$ meV/Å^2). Tab. S3 gives an overview of the different reference chemical potentials.

To determine the bulk transition from Cu to Cu_2O in our surface energy and phase diagrams, we calculate the chemical potential of Cu_2O analog to Cu for the static and dynamic approaches. We show the corresponding data also in Tab. S3.

Table S3: Free energy for the Cu and Cu_2O bulk. For Cu we list the bulk and slab derived free energy.

T [K]	$\frac{G_{\mathrm{Cu,bulk}}(T,p_0)}{N_{\mathrm{Cu}}}$ [eV]	$\frac{G_{\mathrm{Cu,slab}}(T,p_0)}{N_{\mathrm{Cu}}}$ [eV]	$\frac{G_{\mathrm{Cu_2O}}(T,p_0)}{N_{\mathrm{Cu}}}$ [eV]
0*	-3.728	-3.728	-6.818
300	-3.650	-3.651	-6.697
332	-3.642	-3.642	-6.685
366	-3.633	-3.633	-6.671
405	-3.623	-3.623	-6.655
447	-3.611	-3.613	-6.639
494	-3.598	-3.599	-6.620
546	-3.584	-3.585	-6.598
604	-3.568	-3.570	-6.574
667	-3.550	-3.553	-6.548
737	-3.531	-3.534	-6.519
814	-3.509	-3.512	-6.487
900	-3.484	-3.487	-6.450

1.5.4 Frozen surface's energy

Our non-symmetric core slab contains a working surface at which we vary the overlayer composition during our sampling (see Sec. 1.3.1) and a frozen, ideal Cu(111) surface on its backside. In eq. 2, 3, 4, and 7 we subtract the surface energy γ_{core} of this frozen backside to obtain the correct surface energy of the working surface. To derive γ_{core} we require two reference calculations, one of the pristine core Cu(111) slab with the backside frozen (as used in our production calculations) and one of a symmetric Cu(111) slab without frozen atoms. This procedure is analog for static (0 K) and dynamic calculations. In the former case the reference slabs need to be relaxed, in the latter we perform a MD simulation for each temperature.

From the reference calculation of the clean symmetric Cu(111) slab without any frozen atoms, we evaluate the surface free energy γ_{sym} as:

$$\gamma_{\text{sym}}(T,p) = \frac{G_{\text{sym}}(T,p,N_{\text{Cu}}^{\text{sym}}) - N_{\text{Cu}}^{\text{sym}}\mu_{\text{Cu}_{\text{bulk}}}(T,p)}{2A_{\text{surface}}} \tag{16}$$

where G_{sym} is the Gibbs free energy of our symmetric Cu(111) model, $N_{\text{Cu}}^{\text{sym}}$ the number contained Cu atoms and A_{surface} the model's surface area. From the reference calculation of the symmetric Cu(111) slab with frozen atoms we obtain an expression containing γ_{sym} and γ_{core}:

$$\gamma_{\text{sym}}(T,p) + \gamma_{\text{core}}(T,p) = \frac{G_{\text{core}}(T,p,N_{\text{Cu}}^{\text{core}}) - N_{\text{Cu}}^{\text{core}}\mu_{\text{Cu}_{\text{bulk}}}(T,p)}{A_{\text{surface}}} \tag{17}$$

where G_{core} is the Gibbs free energy of our Cu(111) model with fixed backside and $N_{\text{Cu}}^{\text{core}}$ the number contained Cu atoms. We can subtract eq. 16 from 17 to derive an expression for γ_{core}:

$$\gamma_{\text{core}}(T,p) = \frac{G_{\text{core}}(T,p,N_{\text{Cu}}^{\text{core}}) - \frac{1}{2}G_{\text{sym}}(T,p,N_{\text{Cu}}^{\text{sym}}) - (N_{\text{Cu}}^{\text{core}} - \frac{1}{2}N_{\text{Cu}}^{\text{sym}})\mu_{\text{Cu}_{\text{bulk}}}(T,p)}{A_{\text{surface}}} \tag{18}$$

It is important to highlight, that γ_{core} as obtained from eq. 18 contains beside the frozen surface's energy, also a correction for the interaction of the frozen row of Cu atoms with the adjacent relaxed or mobile row of Cu atoms. This correction is relevant for the surface energies obtained from the REMD simulations which would otherwise be nonphysically high.

1.5.5 Vibrational contributions to the inner energy

To improve the comparability of the literature structures with the REMD simulation, we perform phonon calculations within the harmonic approximation using phonopy.[S9] Following the vibrational contributions that are included in the energy average of (RE)MD simulations, we neglect the zero-point energy for our surface models and adjust the inner energy using

only the harmonic heat capacity. Fig. S8 shows the resulting shift in surface free energy from 0 to 600 K. The surface and bulk phase transitions shift to higher potential $\Delta\mu_{\mathrm{O}}$ by ≈ 0.07. The shifted 600 K bulk transition is in line with the REMD predicted bulk transition.

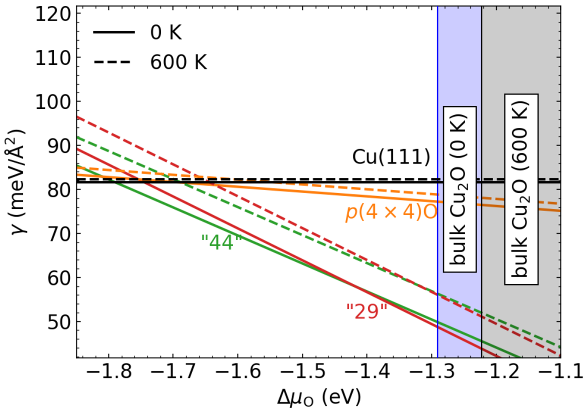

Figure S8: Vibrational influence on the surface free energy γ. The solid lines show γ at 0 K for selected literature models. The corresponding bulk transition is visualized by the blue area. The solid lines show γ, including the vibrational contributions to the inner energy at 600 K, for selected literature models. The corresponding bulk transition is visualized by the gray area.

2 Structural data of sampled ensembles

2.1 Characterization of the −O−Cu−O− network

To characterize the complex two-dimensional −O−Cu−O− networks that form on the Cu(111) surface, we evaluate the expectation value of the contained rings and chains, based on a graph-theory approach (see Sec. 1.4) for the entire ensemble, as a function of the oxygen chemical potential $\Delta\mu_{\mathrm{O}}$. We depict this analysis in Fig. S9a for three different temperatures.

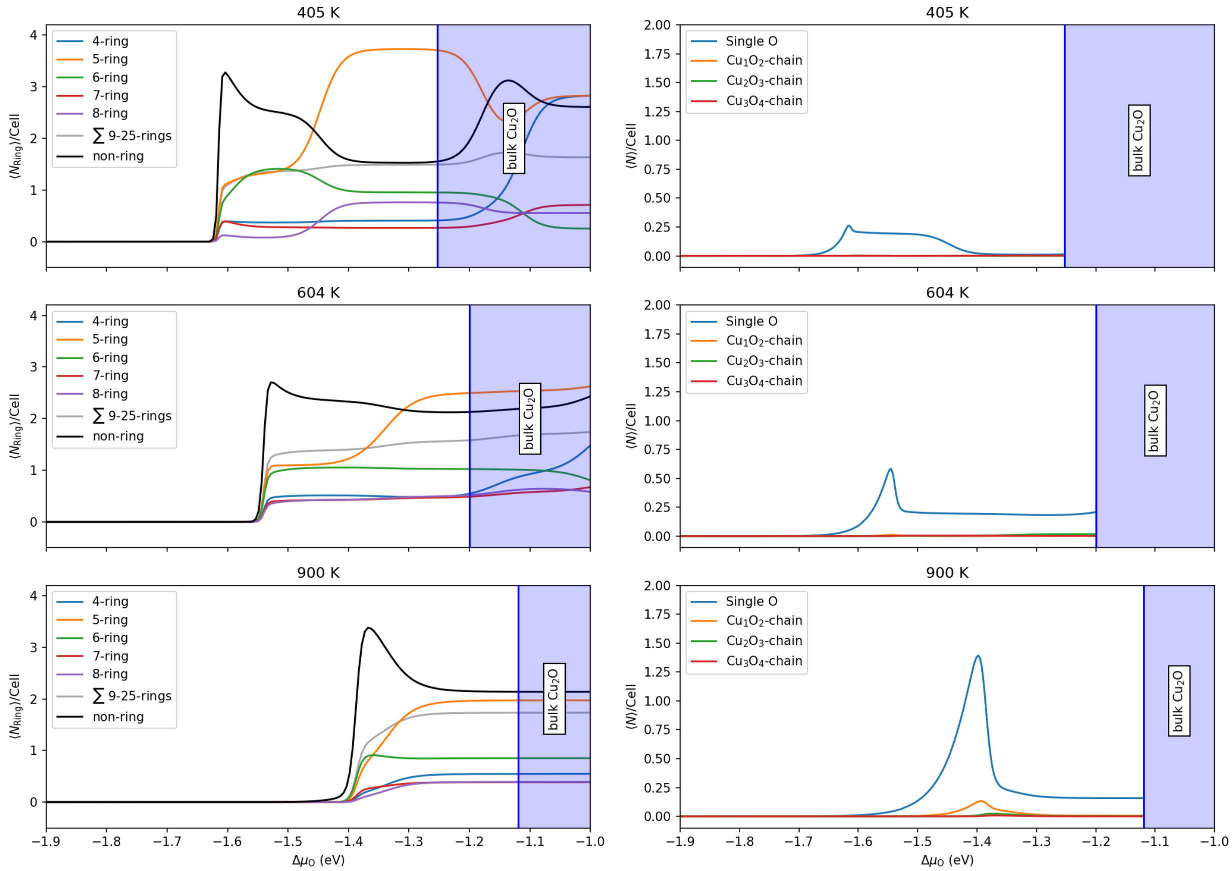

(a) Ring distribution

(b) O* adsorbates, and small isolated $\mathrm{Cu_xO_{x+1}}$ elements

Figure S9: The plots show the expectation value of differently sized rings (a) and O* adsorbates, and small isolated $\mathrm{Cu_xO_{x+1}}$ units (b) vs. O chemical potential $\Delta\mu_{\mathrm{O}}$ for different temperatures.

The network classification gives also access to adsorbed *O and isolated $\mathrm{Cu_xO_{x+1}}$ units

(see Fig. S9b). Both single *O adsorbates, and CuO_2 units appear to be entropically stabilized since they become more relevant with increasing temperature (see blue and orange line in Fig. S9b). The CuO_2 units are characterized by a Cu ad-atom being coordinated by two adjacent O atoms (see Fig. S11a). The coverage of larger Cu_xO_{x+1} units is negligible.

2.2 Metastable configurations

Our sampling approach also yields metastable structures that have little or no relevance from a thermodynamic point of view. So far we have focused on the observables (ring size, *O adsorbates, Cu_xO_{x+1} elements) with respect to $\Delta\mu_O$ (see Fig. S9), capturing solely the thermodynamically relevant configurations. To better visualize the otherwise elusive metastable configurations, we separate our data set in the compositional space and analyze different observables with regard to the O-coverage (θ_O, see Fig. S10). For each θ_O, we obtain the observables from the canonical ensemble with the lowest average energy (see Eq. 6) out of all canonical ensembles with the given θ_O, which differ in the number of additional Cu atoms.

The low-θ_O configurations ($\theta_O \leq 0.16$ ML) are defined by adsorbed *O (blue line in Fig. S10b) in addition to initial CuO_2 units (orange line in Fig. S10b), and fewer extended Cu_2O_3 and Cu_3O_4 units (red & green line in Fig. S10b). Compared to the thermodynamic picture we discussed in the previous section, all Cu_xO_{x+1} elements experience higher intensities in the θ_O space. However, Cu_2O_3 and Cu_3O_4 elements remain scarce relative to CuO_2 units and adsorbed *O.

Metastable precursor configurations for the CuO monolayer can be found at increased coverage (0.16 ML $< \theta_O \leq$ 0.26 ML): The absorbed *O species (blue line in Fig. S10b) vanish and give way to non-ring links (black line in Fig. S10a, i.e. any −O−Cu−O− elements that are not part of a closed ring), which form −O−Cu−O− network-like islands and chains containing few or no rings (see Fig. S11b). From a thermodynamic point of view, these island- and chain-like configurations (0.16 ML $< \theta_O \leq$ 0.26 ML) are negligible in the REMD based

ensemble, even around the oxidation onset (see Tab. S4). Another family of metastable structures are the high coverage configurations ($\theta_O > 0.45$). Noticeable is a maximum of 4-member rings, observable at $\theta_O \approx 0.5$. This motive is characteristic in the metastable "8" structure which can be synthesized via a hyperthermal oxygen molecular beam.[S33] Furthermore, we observe a dominant fraction of 6-rings at $\theta_O > 0.55$ in the lower temperature ensemble, which we can correlate to the crystallization of Cu_2O multilayer structures, as shown exemplarily in Fig. S11c.

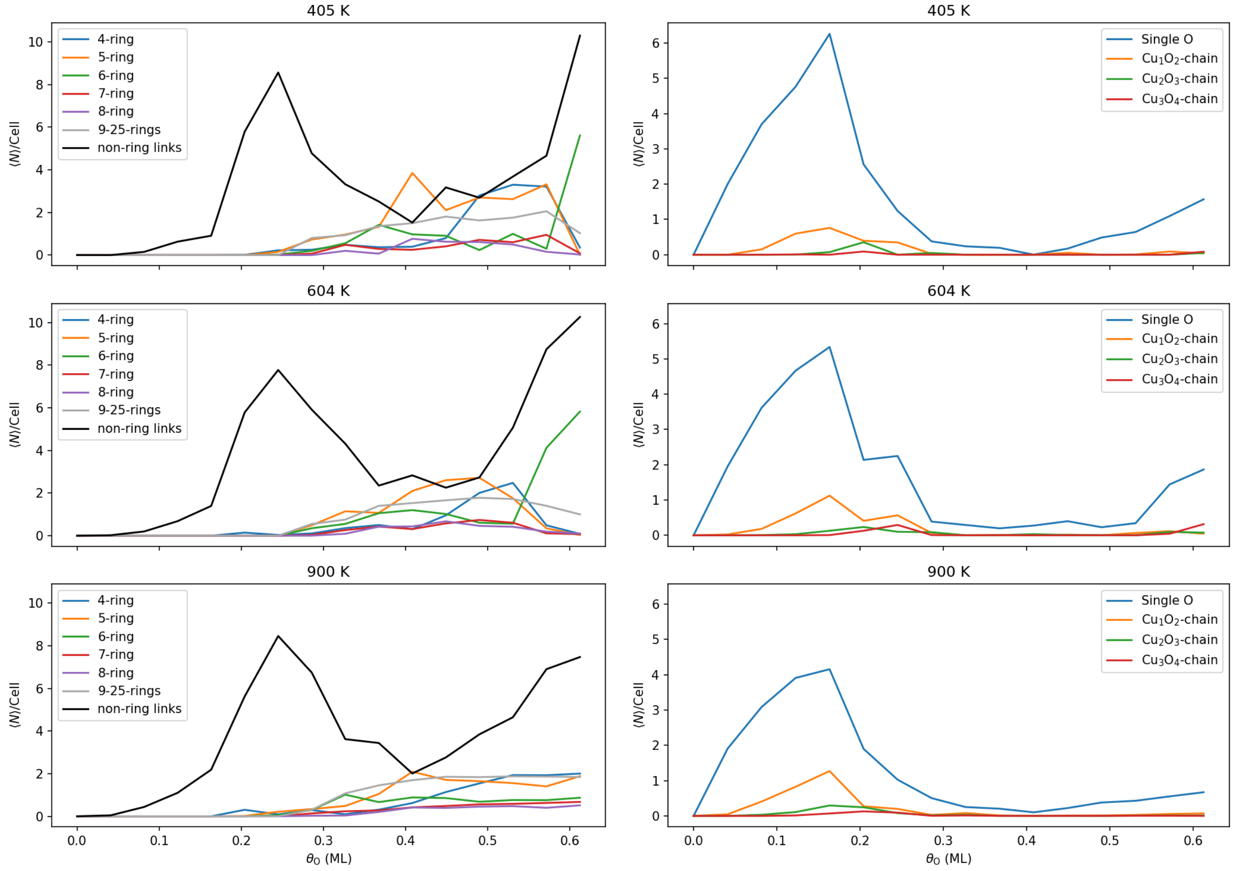

(a) Ring distribution vs. O coverage

(b) Isolated chain size vs. O coverage

Figure S10: The average ring size (a), number of O* adsorbates, and small isolated Cu_xO_{x+1} elements (b) as a function of the O-coverage θ_O for REMD ensembles at different temperatures. For each θ_O and temperature we have multiple canonical ensemble that differ in the amount of Cu atoms. Thus, we calculate the averaged formation energy (See. 1.5.2) of each ensemble at a given θ_O. Subsequently, we take the ensemble with the lowest average formation energy to calculate the determine the expected amount of rings and isolated chains at the given θ_O.

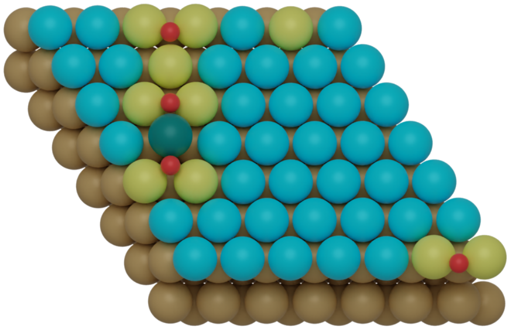

(a) $\theta_O = 0.08$ ML, absorbed oxygen, including a Cu ad-atom being lifted out from the surface by two adjacent O atoms.

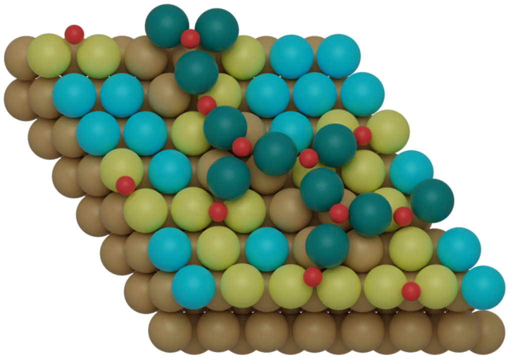

(b) $\theta_O = 0.25$ ML, initial chain formation

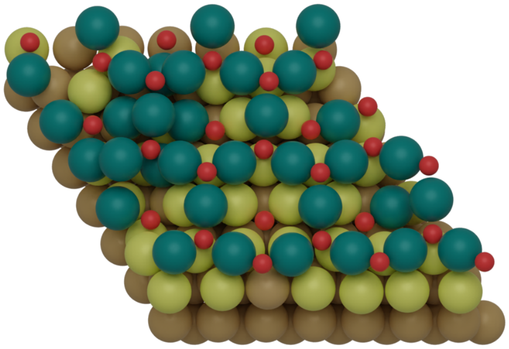

(c) $\theta_O = 0.61$ ML, semi crystalline Cu_2O overlayer

Figure S11: Atomic figures of exemplary low-θ_O and metastable configurations.

2.3 Oxidation onset

To get a more detailed picture of the early oxidation onset, we analyze the composition of the ensemble at $\Delta\mu_O$, where the change in θ_O is at a maximum ($\Delta\mu_O = -1.54$eV at $T = 604$ K, see Fig. 2). For this point in θ_O, we summarize the contributions of various canonical ensembles in Tab. S4. Noticeable is that ensembles with a partially formed $-$O$-$Cu$-$O$-$ network ($\theta_O > 0.16 - 0.29$) are not present, while the clean surface and variations with O_{add} as well as ensembles with "44"-like coverages and fully formed $-$O$-$Cu$-$O$-$ networks

coexist.

Table S4: Coexistence of multiple phases at the surface oxidation onset ($\Delta\mu_O = -1.54$eV) and temperature $T = 604$K. The table shows the probabilities of canonical ensembles (≥ 0.1 %)

θ_O (ML)	Composition	$P_{\text{finite temperature}}$ [%]
0.0	Cu-111	36.65
0.04	O_2@Cu-111	17.2
0.08	O_4@Cu-111	2.2
0.12	O_6@Cu-111	0.1
0.33	$Cu_{18}O_{16}$@Cu-111	4.2
	$Cu_{20}O_{16}$@Cu-111	5.3
	$Cu_{22}O_{16}$@Cu-111	2.4
0.37	$Cu_{22}O_{18}$@Cu-111	31.8
	$Cu_{28}O_{18}$@Cu-111	0.1

2.4 Formal oxidation state of surface Cu atoms

In the previous section, we looked at larger structural motives. To obtain a more detailed picture of the chemical environments on the surfaces, we evaluate the ensemble averages of the CN of Cu-surface atoms as a function of the oxygen chemical potential $\Delta\mu_O$ and θ_O as shown in Fig. S12 a & b respectively.

We exclusively encounter clean Cu surface environments (CN_{Cu-O}=0, gray line in Fig. S12a) for low chemical potentials. As depicted in Fig. S12a, an increase of $\Delta\mu_O$ leads to a slight increase of CN_{Cu-O}=1 environments (blue line) just before the first major phase transition occurs. As discussed in sec. 2.2 the initial CN_{Cu-O}=1 environments are coordinated by adsorbed *O which become more probable at increased temperatures. After the first major phase transition ($\Delta\mu_O \approx -1.62, -1.55, -1.39$ eV for 405, 604, and 900 K), the surface is dominated by CN_{Cu-O}=2 Cu environments that make up the characteristic −O−Cu−O− monolayer (ML), and have the same CN as Cu_2Obulk environments (CN_{Cu-O}(Cu_2O-bulk)=2). In addition to the CN_{Cu-O}=2 Cu atoms, CN_{Cu-O}=1 environments are also present, linking the

$-\mathrm{O}-\mathrm{Cu}-\mathrm{O}$ network to the underlying Cu substrate. While, our data suggest that highly oxidized environments $\mathrm{CN_{Cu-O}}$=4 are not relevant from a thermodynamic perspective as they only show negligible amounts close to the bulk oxidation to Cu_2O (see Fig. S12 b), $\mathrm{CN_{Cu-O}}$=3 environments appear as minority species at elevated temperatures ($\langle N \rangle$/cell ≈ 0.5). Notable is also the finite population of clean Cu-surface environments ($\mathrm{CN_{Cu-O}} = 0$), which can be found at higher temperatures over the full $\Delta\mu_{\mathrm{O}}$-range until the bulk oxidation at high temperatures.

We find higher quantities of $\mathrm{CN_{Cu-O}}$=3 and CuO-like $\mathrm{CN_{Cu-O}}$=4 ($\mathrm{CN_{Cu-O}}$(CuO-bulk)=4) environments at high O coverages $\theta_{\mathrm{O}} > 0.45$. These coverages are meta-stable to Cu_2O-bulk oxidation (compare Fig. 2), which is in agreement with the appearance of CuO-like environments in plasma oxidation and electrochemical studies,[S34,S35] as these metastable states might be kinetically stabilized.

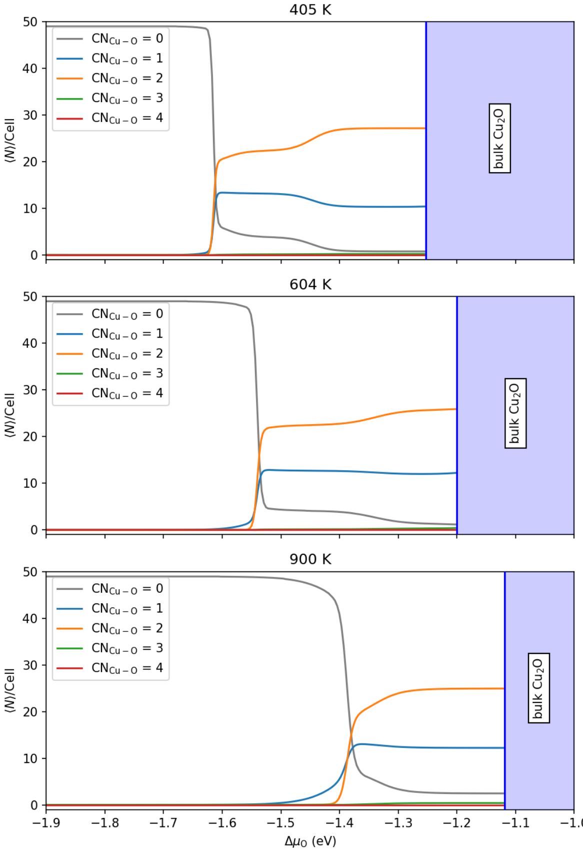

(a) Mean count of zero-, one-, two-, three-, and four-fold O-coordinated (CN) Cu against $\Delta\mu_O$

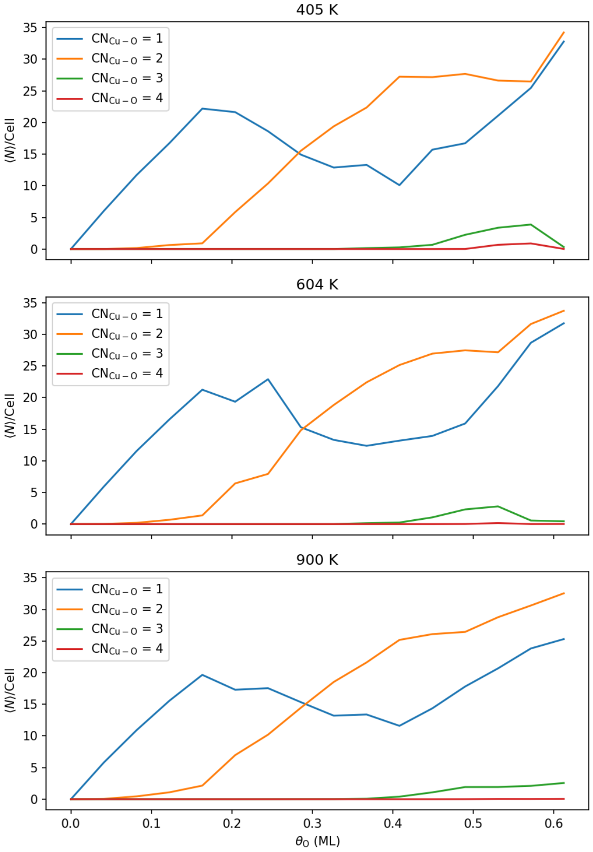

(b) Mean count of one-, two-, three-, and four-fold O-coordinated (CN) Cu against θ_O

Figure S12: Mean count of various O-coordinated (CN) Cu against (a) $\Delta\mu_O$ and (b) θ_O in our REMD-based ensemble at different temperatures. a) visualizes the number of local environments according to the thermodynamic ensemble. b) visualizes the environment count vs θ_O, and thus also reveals structural information about metastable configurations. For each θ_O and temperature in b) we have multiple canonical ensemble that differ in the amount of Cu atoms. Thus, we calculate the averaged formation energy (See. 1.5.2) of each ensemble at a given θ_O. Subsequently, we take the ensemble with the lowest average formation energy to calculate the determine the expected number of environments at the given θ_O.

References

(S1) Kresse, G.; Furthmüller, J. Efficient iterative schemes for ab initio total-energy calculations using a plane-wave basis set. *Phys. Rev. B* **1996**, *54*, 11169.

(S2) Kresse, G.; Joubert, D. From ultrasoft pseudopotentials to the projector augmented-wave method. *Phys. Rev. B* **1999**, *59*, 1758.

(S3) Blöchl, P. E. Projector augmented-wave method. *Phys. Rev. B* **1994**, *50*, 17953.

(S4) Perdew, J. P.; Burke, K.; Ernzerhof, M. Generalized gradient approximation made simple. *Phys. Rev. Lett.* **1996**, *77*, 3865.

(S5) Chiter, F.; Costa, D.; Maurice, V.; Marcus, P. DFT-based Cu (111)—— Cu2O (111) model for copper metal covered by ultrathin copper oxide: structure, electronic properties, and reactivity. *J. Phys. Chem. C* **2020**, *124*, 17048–17057.

(S6) Lee, Y.-J.; Ly, T. T.; Lee, T.; Palotás, K.; Jeong, S. Y.; Kim, J.; Soon, A. Completing the picture of initial oxidation on copper. *Applied Surf. Sci.* **2021**, *562*, 150148.

(S7) Kautek, W.; Gordon, J. G. XPS studies of anodic surface films on copper electrodes. *J. Electrochem. Soc.* **1990**, *137*, 2672.

(S8) Gattinoni, C.; Michaelides, A. Atomistic details of oxide surfaces and surface oxidation: the example of copper and its oxides. *Surf. Sci. Reports* **2015**, *70*, 424–447.

(S9) Togo, A. First-principles phonon calculations with phonopy and phono3py. *Journal of the Physical Society of Japan* **2023**, *92*, 012001.

(S10) Soon, A.; Todorova, M.; Delley, B.; Stampfl, C. Oxygen adsorption and stability of surface oxides on Cu (111): A first-principles investigation. *Phys. Rev. B* **2006**, *73*, 165424.

(S11) Zhu, B.; Huang, W.; Lin, H.; Feng, H.; Palotás, K.; Lv, J.; Ren, Y.; Ouyang, R.; Yang, F. Vacancy Ordering in Ultrathin Copper Oxide Films on Cu (111). *J. Am. Chem. Soc.* **2024**,

(S12) Peterson, A. A.; Abild-Pedersen, F.; Studt, F.; Rossmeisl, J.; Nørskov, J. K. How copper catalyzes the electroreduction of carbon dioxide into hydrocarbon fuels. *Energy & Environmental Science* **2010**, *3*, 1311–1315.

(S13) Bernstein, N.; Csányi, G.; Deringer, V. L. De novo exploration and self-guided learning of potential-energy surfaces. *npj Comput. Mater.* **2019**, *5*, 99.

(S14) Jinnouchi, R.; Miwa, K.; Karsai, F.; Kresse, G.; Asahi, R. On-the-fly active learning of interatomic potentials for large-scale atomistic simulations. *J. Phys. Chem. Lett.* **2020**, *11*, 6946–6955.

(S15) Timmermann, J.; Lee, Y.; Staacke, C. G.; Margraf, J. T.; Scheurer, C.; Reuter, K. Data-efficient iterative training of Gaussian approximation potentials: application to surface structure determination of rutile IrO2 and RuO2. *J. Chem. Phys.* **2021**, *155*.

(S16) Jain, A.; Ong, S. P.; Hautier, G.; Chen, W.; Richards, W. D.; Dacek, S.; Cholia, S.; Gunter, D.; Skinner, D.; Ceder, G.; others Commentary: The Materials Project: A materials genome approach to accelerating materials innovation. *APL materials* **2013**, *1*.

(S17) Ong, S. P.; Richards, W. D.; Jain, A.; Hautier, G.; Kocher, M.; Cholia, S.; Gunter, D.; Chevrier, V. L.; Persson, K. A.; Ceder, G. Python Materials Genomics (pymatgen): A robust, open-source python library for materials analysis. *Comput. Mater. Sci.* **2013**, *68*, 314–319.

(S18) Bartók, A. P.; Payne, M. C.; Kondor, R.; Csányi, G. Gaussian approximation potentials: The accuracy of quantum mechanics, without the electrons. *Phys. Rev. Lett.* **2010**, *104*, 136403.

(S19) Deringer, V. L.; Bartók, A. P.; Bernstein, N.; Wilkins, D. M.; Ceriotti, M.; Csányi, G. Gaussian process regression for materials and molecules. *Chem. Rev.* **2021**, *121*, 10073–10141.

(S20) Batatia, I.; Kovacs, D. P.; Simm, G.; Ortner, C.; Csányi, G. MACE: Higher order equivariant message passing neural networks for fast and accurate force fields. *Adv. Neural Inf. Process. Syst.* **2022**, *35*, 11423–11436.

(S21) Earl, D. J.; Deem, M. W. Parallel tempering: Theory, applications, and new perspectives. *Phys. Chem. Chem. Phys.* **2005**, *7*, 3910–3916.

(S22) Eldar, Y.; Lindenbaum, M.; Porat, M.; Zeevi, Y. Y. The farthest point strategy for progressive image sampling. *IEEE transactions on image processing* **1997**, *6*, 1305–1315.

(S23) Gelžinytė, E.; Wengert, S.; Stenczel, T. K.; Heenen, H. H.; Reuter, K.; Csányi, G.; Bernstein, N. wfl Python toolkit for creating machine learning interatomic potentials and related atomistic simulation workflows. *The Journal of Chemical Physics* **2023**, *159*.

(S24) Schaaf, L. L.; Fako, E.; De, S.; Schäfer, A.; Csányi, G. Accurate energy barriers for catalytic reaction pathways: an automatic training protocol for machine learning force fields. *npj Computational Materials* **2023**, *9*, 180.

(S25) Abraham, M. J.; Gready, J. E. Ensuring mixing efficiency of replica-exchange molecular dynamics simulations. *Journal of Chemical Theory and Computation* **2008**, *4*, 1119–1128.

(S26) Heenen, H. H.; Gauthier, J. A.; Kristoffersen, H. H.; Ludwig, T.; Chan, K. Solvation at metal/water interfaces: An ab initio molecular dynamics benchmark of common computational approaches. *J. Chem. Phys.* **2020**, *152*.

(S27) Lai, K. C.; Matera, S.; Scheurer, C.; Reuter, K. A fuzzy classification framework to identify equivalent atoms in complex materials and molecules. *J. Chem. Phys.* **2023**, *159*.

(S28) McInnes, L.; Healy, J.; Astels, S. hdbscan: Hierarchical density based clustering. *The Journal of Open Source Software* **2017**, *2*.

(S29) Hagberg, A.; Swart, P. J.; Schult, D. A. *Exploring network structure, dynamics, and function using NetworkX*; 2008.

(S30) Rogal, J.; Reuter, K. Ab initio atomistic thermodynamics for surfaces: A primer. *experiment, modeling and simulation of gas-surface interactions for reactive flows in hypersonic flights* **2007**, *14*, 2–1.

(S31) Reuter, K.; Scheffler, M. Composition, structure, and stability of RuO 2 (110) as a function of oxygen pressure. *Phys. Rev. B* **2001**, *65*, 035406.

(S32) Chase, M. W. NIST-JANAF Thermochemical Tables 4th ed. *J. Phys. Chem. Ref. Data.* **1998**, 1529–1564.

(S33) Kim, H. J.; Lee, G.; Oh, S.-H. V.; Stampfl, C.; Soon, A. Recalibrating the Experimentally Derived Structure of the Metastable Surface Oxide on Copper via Machine Learning-Accelerated In Silico Global Optimization. *ACS Nano* **2024**, *18*, 4559–4569.

(S34) Arán-Ais, R. M.; Scholten, F.; Kunze, S.; Rizo, R.; Roldan Cuenya, B. The role of in situ generated morphological motifs and Cu (i) species in C2+ product selectivity during CO2 pulsed electroreduction. *Nat. Energy* **2020**, *5*, 317–325.

(S35) Kunze, S.; Tănase, L. C.; Prieto, M. J.; Grosse, P.; Scholten, F.; de Souza Caldas, L.; van Vörden, D.; Schmidt, T.; Cuenya, B. R. Plasma-assisted oxidation of Cu (100) and Cu (111). *Chem. Sci.* **2021**, *12*, 14241–14253.
